# Operative management of acute abdomen after bariatric surgery in the emergency setting: the OBA guidelines

**DOI:** 10.1186/s13017-022-00452-w

**Published:** 2022-09-27

**Authors:** Belinda De Simone, Elie Chouillard, Almino C. Ramos, Gianfranco Donatelli, Tadeja Pintar, Rahul Gupta, Federica Renzi, Kamal Mahawar, Brijesh Madhok, Stefano Maccatrozzo, Fikri M. Abu-Zidan, Ernest E. Moore, Dieter G. Weber, Federico Coccolini, Salomone Di Saverio, Andrew Kirkpatrick, Vishal G. Shelat, Francesco Amico, Emmanouil Pikoulis, Marco Ceresoli, Joseph M. Galante, Imtiaz Wani, Nicola De’ Angelis, Andreas Hecker, Gabriele Sganga, Edward Tan, Zsolt J. Balogh, Miklosh Bala, Raul Coimbra, Dimitrios Damaskos, Luca Ansaloni, Massimo Sartelli, Nikolaos Parasas, Yoram Kluger, Elias Chahine, Vanni Agnoletti, Gustavo Fraga, Walter L. Biffl, Fausto Catena

**Affiliations:** 1 Department of Emergency, Digestive and Metabolic Minimally Invasive Surgery, Poissy and Saint Germain en Laye Hospitals, Poissy-Ile de France, France; 2 GastroObesoCenter Institute for Metabolic Optimization, Sao Paulo, Brazil; 3 Interventional Endoscopy and Endoscopic Surgery, Hôpital Privé Des Peupliers, Paris, France; 4 Department of Abdominal Surgery, Ljubljana University Medical Centre, Ljubljana, Slovenia; 5 Division of Minimally Invasive Surgery and Bariatrics, Beth Israel Deaconess Medical Center, Boston, MA USA; 6 General Surgery and Trauma Team, ASST Niguarda, Piazza Ospedale Maggiore 3, 20162 Milano, Milan Italy; 7 South Tyneside and Sunderland NHS Foundation Trust, Sunderland, UK; 8 East Midlands Bariatric and Metabolic Institute, University Hospitals of Derby and Burton NHS Trust, Derby, UK; 9 Department of Bariatric Surgery, Istituto Di Cura Beato Matteo, Vigevano, Italy; 10 Department of Surgery, College of Medicine and Health Sciences, UAE University, Al-Ain, United Arab Emirates; 11 Denver Health System - Denver Health Medical Center, Denver, USA; 12 Department of General Surgery, Royal Perth Hospital, University of Western Australia, Perth, Australia; 13 Department of Emergency and Trauma Surgery, Pisa University Hospital, Pisa, Italy; 14 Department of Surgery, Madonna Del Soccorso Hospital, San Benedetto del Tronto, Italy; 15 Department of General, Acute Care, Abdominal Wall Reconstruction, and Trauma Surgery, Foothills Medical Centre, Calgary, AB Canada; 16 Department of General Surgery, Tan Tock Seng Hospital, Singapore, Singapore; 17 Department of Surgery, John Hunter Hospital and The University of Newcastle, Newcastle, MSW Australia; 18 3Rd Department of Surgery, Attikon General Hospital, National and Kapodistrian University of Athens (NKUA), Athens, Greece; 19 General Surgery, Monza University Hospital, Monza, Italy; 20 University of California, Davis 2315 Stockton Blvd., Sacramento, CA 95817 USA; 21 Government Gousia Hospital, Srinagar, Kashmir India; 22 Service de Chirurgie Digestive Et Hépato-Bilio-Pancréatique - DMU CARE, Hôpital Henri Mondor, Paris, France; 23 Department of General and Thoracic Surgery, University Hospital of Giessen, Giessen, Germany; 24 Emergency Surgery and Trauma, Fondazione Policlinico Universitario A. Gemelli IRCCS, Università Cattolica del Sacro Cuore, Rome, Italy; 25 Department of Emergency Medicine, Radboud University Medical Center, Nijmegen, The Netherlands; 26 Department of Traumatology, John Hunter Hospital and University of Newcastle, Newcastle, NSW Australia; 27 Trauma and Acute Care Surgery Unit, Hadassah - Hebrew University Medical Center, Jerusalem, Israel; 28 Riverside University Health System Medical Center, Loma Linda University School of Medicine, Riverside, CA USA; 29 General and Emergency Surgery, Royal Infirmary of Edinburgh, University of Edinburgh, Edinburgh, UK; 30 Department of Surgery, Pavia University Hospital, Pavia, Italy; 31 Department of General Surgery, Macerata Hospital, Macerata, Italy; 32 Division of General Surgery, Rambam Health Care Campus, Haifa, Israel; 33 Department of Emergency and Trauma Surgery, Bufalini Hospital, Cesena, Italy; 34 School of Medical Sciences, University of Campinas (Unicamp), Campinas, SP Brazil; 35 Department of Trauma and Acute Care Surgery, Scripps Memorial Hospital La Jolla, La Jolla, San Diego, CA USA

**Keywords:** Abdominal pain, Bariatric surgery, Acute abdomen, Long-term complication, Emergency surgery, Sleeve gastrectomy, Gastric bypass, Occlusion, Perforation, Bleeding, Peritonitis

## Abstract

**Background:**

Patients presenting with acute abdominal pain that occurs after months or years following bariatric surgery may present for assessment and management in the local emergency units. Due to the large variety of surgical bariatric techniques, emergency surgeons have to be aware of the main functional outcomes and long-term surgical complications following the most performed bariatric surgical procedures. The purpose of these evidence-based guidelines is to present a consensus position from members of the WSES in collaboration with IFSO bariatric experienced surgeons, on the management of acute abdomen after bariatric surgery focusing on long-term complications in patients who have undergone laparoscopic sleeve gastrectomy and laparoscopic Roux-en-Y gastric bypass.

**Method:**

A working group of experienced general, acute care, and bariatric surgeons was created to carry out a systematic review of the literature following the Preferred Reporting Items for Systematic Review and Meta-analysis Protocols (PRISMA-P) and to answer the PICO questions formulated after the Operative management in bariatric acute abdomen survey. The literature search was limited to late/long-term complications following laparoscopic sleeve gastrectomy and laparoscopic Roux-en-Y gastric bypass.

**Conclusions:**

The acute abdomen after bariatric surgery is a common cause of admission in emergency departments. Knowledge of the most common late/long-term complications (> 4 weeks after surgical procedure) following sleeve gastrectomy and Roux-en-Y gastric bypass and their anatomy leads to a focused management in the emergency setting with good outcomes and decreased morbidity and mortality rates. A close collaboration between emergency surgeons, radiologists, endoscopists, and anesthesiologists is mandatory in the management of this group of patients in the emergency setting.

## Background

Obesity is associated with a higher incidence of multiple life-threatening illnesses including diabetes, cardiovascular diseases, and cancer. It is associated with an increased risk of mortality. The World Health Organization reported that in 2016, more than 1.9 billion adults were overweight. Of these over 650 million were individuals living with obesity [https://www.who.int/news-room/fact-sheets/detail/obesity-and-overweight].

Bariatric surgery is the most effective treatment for severe and complex obesity. Not surprisingly, the number of bariatric surgical procedures performed has increased recently [1]. These procedures are relatively “young” and have been refined by experience and studies on long-term outcomes. They include ultra-specific upper gastrointestinal techniques for re-creating new intestinal anatomy with proper physiology (metabolic surgery).

Procedure selection depends on patient’s individual factors such as body mass index, comorbidities, commitment to postoperative follow-up attendance, understanding of potential surgical risks and complications, the patient’s choice, and multidisciplinary team assessment.

The International Federation for Surgery of Obesity and metabolic disorders (IFSO) reported that internationally the laparoscopic sleeve gastrectomy (LSG) is the most performed surgical procedure, followed by laparoscopic Roux-en-Y gastric bypass (LRYGB), one anastomosis gastric bypass (OAGB), and laparoscopic adjustable gastric banding (LAGB) [1].

Usually patients with early postoperative complications are managed by bariatric surgeons during the hospital postoperative stay or in the short mid-period follow-up. Nevertheless, patients with acute abdominal pain (AAP) that occurs after months or years following surgery may present for assessment and management in the local emergency units. Moreover, at least 2% of worldwide bariatric procedures are provided for “medical tourists” moving from waiting lists or unaffordable bariatric healthcare costs. Several countries including Mexico, Lebanon, and Romania dominate with established providers caring for individuals living with obesity, mainly from the USA, the UK, and Germany. Unfortunately, this group of patients does not have an adequate follow-up and, if not well informed, is at high risk to present with long-term complications [2].

Complications following surgical treatment of severe obesity vary depending on the procedure performed and can be as high as 40% [3].

Due to the large variety of surgical bariatric techniques, the functional outcomes and long-term surgical complications remain not well understood and under-reported.

A recent international web survey of the World Society of Emergency Surgery (WSES) [4] assessed the emergency surgeon’s point of view in managing long-term surgical complications from bariatric surgical procedures and highlighted the necessity of providing evidence-based recommendations for non-bariatric emergency surgeons.

The purpose of these guidelines is to present a consensus position from members of the WSES in collaboration with IFSO experienced members, on the management of acute abdomen after bariatric surgery focusing on long-term complications in patients who undergone LSG and LRYGB.

## Methodology

According to the Operative management in Bariatric Acute abdomen (OBA) Survey outcomes [4], the principal investigator identified research areas and main topics to investigate about the management of acute abdomen after bariatric surgery and developed PICO questions [5].

A working group of experienced general, acute care, and bariatric surgeons was created to carry out a systematic review of the literature in accordance with the Preferred Reporting Items for Systematic Review and Meta-analysis Protocols (PRISMA-P) [6] and to answer the PICO questions by the WSES steering committee by searching the MEDLINE (PubMed), Embase, and Scopus databases.

Every effort was made to provide a comprehensive assessment of the published evidence.

Literature search was limited to LSG and LRYGB.

All available articles (systematic reviews, randomized controlled trials, meta-analysis, epidemiological studies, case series, and survey outcome reports) which focused on surgical emergencies related to previous surgical bariatric procedures and published in the English language were included in the analysis.

A summary of evidence, statements, and recommendations was developed in accordance with the GRADE methodology [7] and is reported in Table 1.

The principal investigator supervised each step of literature searching, article selection, and the final presentation of evidence.

A late or long-term complication was defined a postoperative complication occurring 4 weeks or more after the bariatric surgery.

“Bariatric patient” was defined as a patient who undergone bariatric surgery in the past.

The provisional statements and the supporting literature were reviewed and discussed by email and modified as necessary. Controversies in statements and recommendations have been agreed on using the Delphi method. The first draft of the paper was submitted to the WSES scientific international board that consisted of emergency and acute care surgeons, anesthesiologists, emergency physicians, radiologists, infection disease physicians, and qualified nursing personnel for evaluation and approval.

**Fig. 1:**
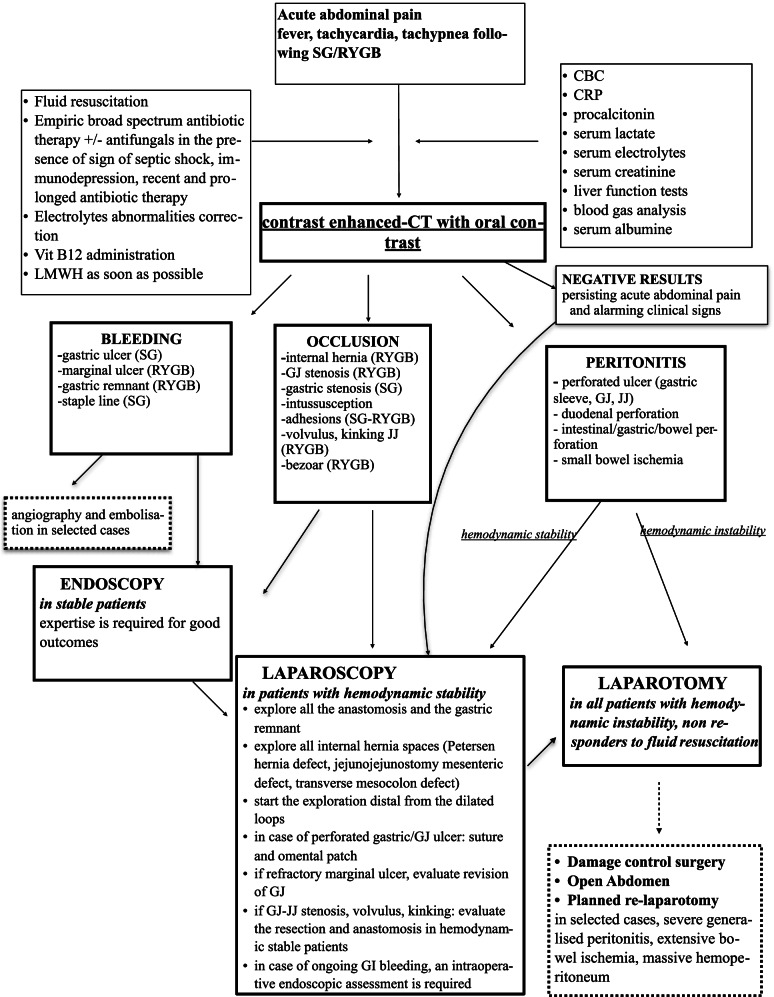
Decision making algorithm for the management of acute abdomen after bariatric surgery. SG: sleeve gastrectomy, RYGB: Roux en Y gastric bypass; GJ: gastrojejunostomy; JJ: jejunojenostomy; LMWH: low molecular weight heparin; CBC: complete blood count cells; GI: gastrointestinal; CT: computed tomography

A decision-making algorithm for the assessment and management of patients presenting with acute abdominal pain after bariatric surgery was developed and is provided in Fig. 1.

### Notes on the use of the guidelines

These guidelines are the result of an extensive review of the literature with the formulation of recommendation based on the evidence. The practice guidelines promulgated in this work do not represent a standard of practice. These are suggested plans of care, based on best available evidence and the consensus of experts, but they do not exclude other approaches as being within the standard of practice. These guidelines should be tailored by the treating surgeons and individualized for each patient depending on the setting and should not be followed blindly.

## Results

### Q.1. WHICH ARE THE “ALARMING" CLINICAL SIGNS AND SYMPTOMS FOR ACUTE SURGICAL ABDOMEN IN PATIENTS WITH A PREVIOUS HISTORY OF BARIATRIC SURGERY?

#### Statement 1.1

Tachycardia ≥ 110 beats per minute, fever ≥ 38 °C, hypotension, respiratory distress with tachypnea and hypoxia, and decreased urine output are alarming clinical signs in patients presenting with acute abdominal pain with a previous history of bariatric surgery **(QoE: low)**.

#### Statement 1.2

In the presence of respiratory distress and hypoxia, a pulmonary embolism must be systematically excluded **(QoE: low)**.

#### Statement 1.3

In the absence of fever and other signs of sepsis but in the presence of tachycardia (be aware of patients treated with beta blockers) and acute abdominal pain, patient requires immediate laboratory tests and imaging assessment for early and long-term complications following bariatric surgery **(QoE: low)**.

#### Statement 1.4

In the emergency setting, the combination of fever, tachycardia, and tachypnea are significant predictors of an anastomotic leak or staple line leak after sleeve gastrectomy and Roux-en-Y gastric bypass **(QoE: low)**.

#### Statement 1.5

Persisting vomiting and nausea are alarming clinical signs due to the high probability of complications such as internal hernia, volvulus, gastrointestinal stenosis, intestinal ischemia, or marginal ulcer after bariatric surgery **(QoE: low)**.

#### Statement 1.6

The most common clinical presentation of internal hernia after laparoscopic Roux-en-Y gastric bypass is acute onset, persistent crampy/colicky abdominal pain, mostly located in the epigastrium **(QoE: low)**.

#### Statement 1.7

The triad of persistent epigastric pain, pregnancy, and a history of laparoscopic Roux-en-Y gastric bypass should be warning signs for the prompt evaluation of the patient for the high suspicion of internal hernia **(QoE: low)**.

#### Statement 1.8

Any clinical signs of intestinal bleeding such as hematemesis, melena, and hematochezia after bariatric surgery are predictors signs of intra-abdominal complications **(QoE: low)**.

#### Recommendation/1

There are no absolute alarming clinical signs/symptoms for long-term complications after bariatric surgery. Clinical presentation can be non-specific. Any new onset abdominal symptoms should give rise to suspicion for late complications after bariatric surgery.

We recommend against delaying prompt diagnostic work-up and laparoscopic surgical exploration in patients with a previous history of bariatric surgery, presenting with persistent abdominal pain and/or gastrointestinal symptoms, associated with fever, tachycardia, and tachypnea **(Strong recommendation based on low level of evidence 1C)**.

#### Discussion of evidence

Clinical signs and physical examination of patients who have undergone bariatric surgery presenting with AAP can be atypical, insidious, and often resulting in delayed management due to inconclusive clinical and radiological findings, with consequent poor outcomes and high morbidity and mortality rate.

Tachycardia is considered the main alarming sign in the early postoperative period [8]. Late postoperative complications can be revealed by hemodynamic instability, respiratory failure, or renal dysfunction, in the presence of sepsis. However, these signs may not always be present.

Several studies confirmed that acute and chronic abdominal pain is one of the most common and sometimes frustrating consequences after bariatric surgery. Some authors reported that 15–30% of these patients will visit the emergency room or require admission within three years after the bariatric surgical procedure especially LRYGB [9].

Correlation between the anatomical reconstruction and physiological effects and long-term outcomes of different bariatric surgical procedures are not yet fully understood. The altered physiology of various organs dictates a critical assessment of the patient and knowledge of the altered physiological response. This enables earlier detection of complications after surgery and prompt action [10]

In several case series, the most common symptoms identified in diagnosing an anastomotic leak after LSG were abdominal pain, tachycardia, and fever [8, 9].

Rapid weight loss has been associated with internal hernia (IH). Geubbels et al. [11] retrospectively analyzed data on 1583 LRYGB patients presenting with an abdominal pain. Forty patients (2.5%) had IH at explorative laparoscopy. In addition, it was reported that the median time between LRYGB and first onset of IH symptoms was 9 months (range 0–32). Ninety percent of all IH developed within 20 months. Most patients presented with complaints at regular checkups at the outpatient clinic (60%). All patients presented with abdominal complaints, mostly with an acute onset (80%), cramping/colicky nature (65%), and located in the epigastrium (45%). Laboratory studies were performed in majority of the patients, but did not reveal any major pathology.

Santos et al. [12] analyzed 38 patients during the postoperative period of LRYGB who presented with clinical manifestations suggestive of IH after an average of 24 months following the bariatric procedure. All patients presented with pain, 23 presented abdominal distension, 10 had nausea and 12 had vomiting; three of them had dysphagia, three had diarrhea and one had gastroesophageal reflux. The patients had symptoms for an average (range) of 15 (3–50) days. Seventeen (45.9%) patients were seen once, while the other 20 (54.1%) went to the emergency room twice or more.

More than 70% of the patients choosing weight loss surgery were females in child-bearing age [13].

Weight loss improves fertility in women. Nevertheless, pregnancy after LRYGB can increase the risk of IH and intestinal obstruction from associated increased intra-abdominal pressure during pregnancy.

Dave et al. [14] carried out a systematic review, including 27 articles, with a total of 59 patients, regarding internal herniation during pregnancy after LRYGB and showed that epigastric pain, nausea, and vomiting were the most common symptoms. In terms of serum and blood laboratory tests, white blood count was found to be normal in 22 out of 32 (68.75%) reported cases. Serum lactate levels were also found to be normal in 18 out of 20 (90%) of reported cases.

Torres-Villalobos et al. [15] reported in a systematic review that small-bowel obstruction (SBO) in pregnant women presents with signs and symptoms that are commonly found during pregnancy. Vomiting is uncommon after LRYGB because there is no large reservoir to accumulate secretions. The lack of specific signs and symptoms in this group of patients may lead to delayed diagnosis and intervention with an increase in overall morbidity. The evaluation of the post-RYGB pregnant patients with abdominal pain should include a detailed history, physical examination, laboratory work-up, and imaging work-up. Early involvement by the bariatric surgeon optimizes clinical outcomes, but it is not always possible, especially at night.

The OBA survey [4] reported that acute care and emergency surgeons are not confident in managing patients with a previous history of bariatric surgical procedure because of insidious clinical features. This survey, based on the personal experience of 117 international acute care and emergency surgeons, showed that the most common symptoms in the emergency presentation were generalized abdominal pain, vomiting, localized abdominal pain, and abnormal stools transit.

Another on line survey [https://www.1ka.si/admin/survey/index.php?anketa=286953&a=data] was done on members of various surgical bariatric associations. They considered tachycardia to be the most sensitive sign associated with surgical complications after bariatric surgery in the early postoperative period, and as a clinical alarm to closely investigate emergency surgical long-term complications.

Moreover, the combination of fever, tachycardia, and tachypnea in the emergency setting was identified as a significant predictor of an anastomotic leak or staple line leak [16].

Clinical examination of patients with obesity can be unreliable due to body habitus and the absence of the classic signs of peritoneal irritation. This means that postoperative tachycardia should be taken as a serious warning sign of surgical complications after bariatric surgery [8]. The classic signs of peritoneal irritation are usually absent [17].

In the presence of fever, hypotension, tachycardia, tachypnea associated with hypoxia and decreased urine output, signs of shock, and multi-organ failure, a surgical exploration is mandatory without delay. Knowledge of the type of surgery performed may indicate the specific cause of septic complications [18].

### Q.2. WHICH ARE THE MOST SENSITIVE AND SPECIFIC LABORATORY TESTS FOR DIAGNOSIS IN PATIENTS WITH A PREVIOUS HISTORY OF BARIATRIC SURGERY PRESENTING WITH ACUTE ABDOMEN?

#### Statement 2.1

A detailed history, physical examination, laboratory tests, and imaging modalities are mandatory in decision-making algorithm for patients presenting with acute abdominal pain after a previous bariatric surgery, in the emergency setting **(QoE: low)**.

#### Statement 2.2

Laboratory tests including complete blood count cells, serum electrolytes, C-reactive protein (CRP), procalcitonin, serum lactate levels, renal and liver function tests, serum albumin, and blood gas analysis are helpful in the emergency department assessment of this group of patients presenting with acute abdominal pain **(QoE: low)**.

#### Statement 2.3

High CRP level is predictive of both early and late postoperative complications after bariatric surgery **(QoE: low)**.

#### Statement 2.4

CRP has a remarkably higher sensitivity and specificity than white blood count cells or neutrophil count to rule out an abdominal surgical disease. However, a normal CRP level alone does not rule out the possibility of a postoperative complication following a bariatric surgical procedure **(QoE: low)**.

#### Statement 2.5

Elevated serum lactates should not be used as a single marker to exclude internal herniation, because it can occur late in the presence of intestinal ischemia **(QoE: low)**.

#### Statement 2.6

Nutritional deficiencies in vitamins, minerals, and trace elements may follow bariatric surgery and are associated with clinical manifestations and diseases, including anemia, ataxia, hair loss, and Wernicke encephalopathy **(QoE: low)**.

#### Recommendation/2

There is not a biological marker for the diagnosis of long-term complications of bariatric surgery.

We suggest performing a combination of complete blood count cells, serum electrolytes, serum albumin, liver and renal function tests, CRP, procalcitonin and serum lactate levels, blood gas analysis in assessing late complications following bariatric surgery in the emergency setting **(Weak recommendation based on low level of evidence 2C)**.

We suggest considering high CRP level and leukocytosis as predictors of abdominal emergencies following bariatric surgery **(Weak recommendation based on low level of evidence 2C)**.

We suggest assessing the nutritional status of patients undergoing bariatric procedures, including Vitamin D, folic acid, B12, B6, and B1 serum level, because of the high risk of vitamin B complex deficiency and malnutrition **(Weak recommendation based on low level of evidence 2C)**.

#### Discussion of evidence

Clinical examination of patients with a previous history of bariatric surgery presenting with acute abdomen is challenging because of faded clinical symptoms and often chronic abdominal pain. Nevertheless, a detailed history, physical examination, laboratory tests, and imaging modalities are mandatory in decision-making algorithm in emergency setting.

Concerning laboratory tests, the OBA survey reported that a combination of complete blood cell count, serum electrolytes, C-reactive protein (CRP), and procalcitonin (PCT) concentrations are advised in ED [4].

Several meta-analyses and systematic reviews confirmed that the CRP has a high predictive value for postoperative complications in the early postoperative period after abdominal surgery [19, 20]. Several meta-analyses confirmed the usefulness of CRP level in the early diagnosis of postoperative infectious complications after bariatric procedures [21, 22]. In addition, it was demonstrated that bariatric surgery patients with elevated postoperative CRP level are at increased risk for 30-day complications. The low sensitivity of a CRP ≥ 5 mg/dL suggests that a normal CRP level alone does not rule out the possibility of a postoperative complication. However, with its high specificity, there should be an elevated clinical suspicion of a postoperative complication in patients with a CRP ≥ 5 mg/dL [23]. Several studies indicated that CRP is a useful negative predictive test for the development of anastomotic leakage and in detecting abscess formation after LSG and colorectal surgery with remarkably higher sensitivity and specificity than WBC or neutrophil count [24, 25].

PCT levels increase in bacterial infections, rising early in the course of infection, making it a useful biomarker for decision making regarding initiation of antibiotic therapy and management of sepsis when the results of blood culture are not available. Several studies systematically evaluated the clinical value of PCT and CRP in the diagnosis of adult patients with sepsis which demonstrated a higher diagnosis accuracy and specificity of PCT than CRP [26, 27].

A cross sectional study [28] was conducted to compare the performance of PCT, CRP, leukocyte count and lactate levels compared to blood culture in critically ill patients admitted with suspicion of sepsis. One hundred-twenty six patients were studied and it was reported that leukocyte count, CRP and lactate levels were higher in blood culture positive patients but the difference was not significant despite finding that PCT had higher negative predictive value in ruling out bacterial infection.

PCT dosage has its own limitations. It is expensive compared to CRP and WBC, and may falsely rise in cases of acute respiratory distress syndrome, chemical pneumonitis and severe falciparum malaria [29]. Nevertheless, it may have an important role in guiding the antibiotic therapy decision making and de-escalation [30].

Ambe et al. [31] reported that leukocytosis was found in 31.25%, and elevated serum lactate levels in 10% of patients having gastrointestinal obstruction and internal herniation. Therefore, leukocytosis and elevated serum lactate should not be used as markers for internal herniation. An explanation of these findings could be that multi-visceral involvement and ischemia needs to occur to increase systemic lactate. Furthermore, the amount of released lactate from ischemic regions of the bowel needs to exceed the metabolic capacity of the liver through the Cori cycle [32, 33].

People undergoing bariatric surgery are at high risk of developing neurological, cognitive and mental disabilities and cardiovascular disease due to deficiency of vitamin B. Early detection is important to prevent complications including Wernicke encephalopathy, peripheral neuropathy and bariatric beri beri [34, 35]. Vitamin B12 could be administered orally, intra-muscularly, intranasal, intravenous. In the emergency setting, the IV administration is preferred.

### Q.3: WHICH IS THE MOST SPECIFIC AND SENSITIVE RADIOLOGICAL STUDY FOR DIAGNOSIS IN ASSESSING PATIENTS AFTER BARIATRIC SURGERY PRESENTING WITH ABDOMINAL PAIN?

#### Statement 3.1

The diagnostic value of imaging after bariatric surgery depends mostly on the knowledge of the anatomic changes and of the potential complications following bariatric surgery **(QoE: low)**.

#### Statement 3.2

Contrast-enhanced CT scan with oral contrast is the study of choice in patients with a previous history of bariatric surgery presenting with acute abdomen **(QoE: moderate)**.

#### Statement 3.3

Plain abdominal X-ray has a limited role, when CT scan is not available, in detecting bowel distension or/and fluid levels **(QoE: low)**.

#### Statement 3.4

Point-of-care ultrasound can be used by emergency physicians to rule out cholecystitis and biliary diseases, acute appendicitis, and the presence of free intraperitoneal fluid **(QoE: low)**.

#### Statement 3.5

The administration of oral and intravenous contrast is fundamental to find landmarks for the interpretation of images **(QoE: low)**.

#### Statement 3.6

In a pregnant woman with a history of bariatric surgery, US and magnetic resonance imaging (MRI) are preferred to assess acute abdominal pain with the aim of limiting ionizing radiation exposure. Low-dose CT could be performed in very selected cases **(QoE: low)**.

#### Statement 3.7

Diagnostic laparoscopy has higher sensitivity and specificity than any radiological assessment **(QoE: moderate)**.

#### Statement 3.8

The role of angiography and angioembolization in patients presenting with a gastrointestinal bleeding after bariatric surgery is marginal. They could be a valid tool to achieve bleeding control, in selected cases **(QoE: very low)**.

#### Recommendations/3

We recommend the use of contrast-enhanced computed tomography with oral contrast in the assessment of acute abdomen after bariatric surgery, whenever possible. The absence of oral and intravenous contrast can significantly decrease sensitivity and specificity of radiological assessment **(Strong recommendation based on low level evidence 1C)**.

We recommend assessing the acute abdomen in a pregnant woman by US and MRI to limit radiation exposure, though low-dose CT can be useful in selected cases **(Strong recommendation based on low level evidence 1C)**.

We recommend against delaying laparoscopic exploration if there is a high index of clinical suspicion and in the presence of alarming clinical signs/symptoms, even in situations of negative radiological assessment **(Strong recommendation based on low level evidence 1C)**.

#### Discussion of evidence

An accurate and early diagnosis is the cornerstone of the management of all patients presenting with AAP. Misdiagnosis or delay in diagnosis in patients with a previous history of bariatric surgery can have lethal consequences.

The clinical examination could be notoriously unreliable in this group of patients who often had high weight loss after a bariatric surgical procedure (excess of skin and flaccid abdomen, the absence of guarding sign, and abdominal rigidity).

Experienced emergency surgeons are aware that close monitoring and early diagnostic surgical intervention are mandatory in the management of bariatric surgery patients having persistent abdominal pain, even if stable [4]. Diagnostic radiological preoperative accuracy may influence the timing of the surgical exploration, the surgical technique and the outcomes.

The OBA survey showed that radiological exams performed to assess the acute abdomen after bariatric surgery were very useful in the decision making. This included a plain abdominal X-ray (AXR) and a contrast-enhanced computed tomography (CT) for 41.9% (49/117) of emergency surgeons; an abdominal CT with gastrointestinal opacification for 41.9% (49/117) of emergency surgeons, and AXR in standing position and abdominal ultrasound (US) for 13.7% (16/117) of emergency surgeons [4]. The diagnostic value of imaging studies depends mostly on the careful interpretation of the new anatomical landmarks and on the knowledge of the potential complications following bariatric surgery.

AXR has a limited role when a CT scan is not available. It may reveal bowel distension or/and fluid levels. Specific indications for ordering a plain radiography in assessing acute include suspicion of perforated viscus, urinary tract stones, bowel obstruction, and an ingested foreign body [36].

Point-of-care ultrasound (POCUS) is useful in the evaluation of gallbladder pathology, acute appendicitis, free fluid, or intestinal distention.

The contrast-enhanced CT with oral contrast administration is the study of choice in patients with a previous history of bariatric surgery [37–39].

In several studies, the new radiological anatomy in this group of patients at CT was described. The administration of oral and IV contrast is fundamental to identify landmarks for the interpretation of findings [40–45]. If this is not possible in case of allergy to IV or oral hydrosoluble contrast or acute kidney failure, then laparoscopic exploration is mandatory due to the low sensitivity of radiological studies.

After LRYGB, the identification on CT of the gastric pouch, gastro-jejunal anastomosis, jejunal Roux limb, jejuno-jejunal anastomosis, and biliopancreatic limb is essential for detecting potential complications such as internal hernias (IH), small-bowel obstruction (SBO), anastomotic stenosis, perforation, and gastro-gastric fistula.

In LRYGB, oral contrast administered just prior to image acquisition helps to differentiate the gastric pouch and Roux limb from the excluded stomach and biliopancreatic limb, which are not opacified. The Roux limb should be followed along its antecolic or retrocolic course to the jejuno-jejunal anastomosis, typically in the left mid-abdomen. The excluded stomach should be visualized on CT images and is normally collapsed [41].

According to CT scan findings, SBO following LRYGB is classified based on the features of the Roux-alimentary limb, biliopancreatic limb and the involvement of the common distal channel [46].

Moreover, the abdominal CT is an important tool in diagnosing internal herniation, with a high specificity 87.1 (81.7–91.2) % and a high negative predictive value 96.8 (92.9–98.7) % [38].

Garza et al. [47] examined 1000 LRYGB patients for signs of internal hernia (IH). Of the 34 patients that had an IH, 22 (64%) had signs of IH in the CT scan.

Geubbels et al. [11] reported that 40% of the IH patients had signs of an IH on CT scanning. Therefore, a negative CT scan should not rule out an IH.

Agaba et al. [48] suggested that the work-up for LRYGB patient presenting with abdominal complaints should be as follows: Those presenting with acute signs of SBO (vomiting, acute abdomen) should be considered a surgical emergency and require immediate diagnostic laparoscopy. A low threshold for elective re-laparoscopy should be set for these patients to prevent small-bowel ischemia. The algorithm presented by Agaba et al. included an abdominal CT.

After LSG, CT scan is the best radiological exam to diagnose abscesses, perforations, staple line dehiscence, and other complications such as splenic injury or infarction [41].

Alharbi et al. [49] retrospectively analyzed data of 152 consecutive patients who underwent CT for suspected post-SG gastric leak and reported that CT findings sensitivity and specificity of perigastric collection without oral contrast leak were 61% and 88.8%, for oral contrast leak were 28% and 100%, and for gas leak were 10% and 77.7%, respectively. Therefore, indirect sign such as perigastric fluid collection without contrast leak and with variable wall enhancement and extra-luminal gas are the most common CT findings of post-sleeve gastrectomy gastric leak.

In pregnant women with a history of bariatric surgery, US and magnetic resonance imaging (MRI) are preferred to assess acute abdominal pain with the aim of limiting radiation exposure to the embryo or fetus.

Specific sonographic findings associated with SBO include diameter of the small bowel > 25 mm, small-bowel wall edema, “to and fro” peristalsis, free intra-abdominal fluid, and the presence of a sonographic transition point (defined as the location between dilated small bowel proximal to the obstruction and decompressed small bowel distal to the obstruction) despite the gravid uterus [50].

MRI may be considered an alternative to CT which eliminates the risk of radiation exposure of the embryo or fetus. One of the limitations of MR imaging is the use of gadolinium. The current American College of Radiology guidance document for safe MRI practices suggests that MRI contrast agents should not be routinely used in pregnant patients and this decision should be made on a case by case basis accompanied by a risk–benefit analysis. The restricted availability of MRI imaging limits its utility in the emergency setting [51–54].

The radiation exposure of a CT scan is a major concern and the risks and benefits should be evaluated. The absolute risks of fetal effects, are small at doses of 100 mGy and negligible at doses of less than 50 mGy [55]. CT examinations of the abdomen and pelvis rarely exceed 25 mGy. Because the dose from a single-acquisition CT examination of the abdomen and pelvis poses a small risk to fetal health, CT may be appropriate depending on the clinical situation [15–56]. However, an attempt should be made to perform this test with the lowest dose possible. The risks associated with radiation exposure of the conceptus during the second and third trimesters of pregnancy are reduced and also should be considered [55].

In pregnant LRYGB patients, the evaluation of the CT scan by a radiologist experienced in bariatric surgical procedures and by a bariatric surgeon is advised to improve the diagnostic accuracy in detecting IH [15]. Signs on CT that suggest an IH depend on the location. Clustering and crowding of dilated small-bowel loops and congestion may be seen in most of the cases.

The most distinct radiological finding in patients with IH is the “whirlpool sign” that is the swirled appearance of the mesenteric vessels. When the herniated bowel passes through the transverse mesocolon defect, it is located behind the stomach remnant and may produce a mass effect on its posterior wall. If the herniated bowel passes through the jejuno-jejunostomy defect, it is pressed against the abdominal wall, causing central displacement of the colon, with no overlying omental fat. In cases of a Petersen-type hernia, the radiologic diagnosis can be difficult and the only clue may be engorgement and crowding of the mesenteric vessels and evidence of small-bowel obstruction [15].

### Q.4: WHAT IS THE ROLE OF ENDOSCOPY IN THE DIAGNOSIS AND TREATMENT OF LONG-TERM COMPLICATIONS FOLLOWING BARIATRIC SURGERY IN THE EMERGENCY SETTING?

#### Statement 4.1

After contrast-enhanced computed tomography with oral contrast administration, the endoscopic evaluation is the first option to be considered for the diagnosis and management of leaks and fistulae related to bariatric surgery in stable patients **(QoE: moderate)**.

#### Statement 4.2

The endoscopic evaluation should be performed by an expert endoscopist aware of the new anatomy resulting from different surgical bariatric procedures **(QoE: moderate)**.

#### Statement 4.3

The endoscopic management of leaks and fistulae related to bariatric surgery in stable patients is effective and safe when performed in expert centers **(QoE: moderate)**.

#### Statement 4.4

Several endoscopic devices and techniques are available to manage bariatric surgery complications, and they include internal drainage techniques and vacuum therapy, self-expanding metal (SEMS) and plastic stents, clipping techniques, including the use of through-the-scope clips (TTSC) and over-the-scope clips (OTSC), tissue sealants, suturing systems (OverStitch System®) **(QoE: moderate)**.

#### Statement 4.5

The endoscopic internal drainage by pigtail plastic stents is considered an effective alternative to fully covered SEMS in the treatment of late leaks and fistulae after LSG or RYGB providing higher success rates, shorter treatment duration, and lower adverse events rates compared to stenting **(QoE: moderate)**.

#### Statement 4.6

In the presence of gastric stricture after LSG or of anastomotic stricture after LRYGB, the endoscopic dilation performed by achalasia balloon in LSG or through-the-scope dilation hydrostatic balloon for LRYGB should be considered as the first-line treatment **(QoE: low)**.

#### Statement 4.7

Early endoscopic assessment should be performed in case of suspected active intra-gastric bleeding related to a marginal or stomal ulcer in stable patients. **(QoE: low)**.

#### Statement 4.8

Hemostasis of a bleeding ulcer, performed with injection of epinephrine or mechanical hemostasis with endoscopic clips and rubber band ligation are preferred over thermal hemostasis **(QoE: moderate)**.

#### Statement 4.9

Novel hemostatic powders may be considered as a valid therapeutic option in selected patients presenting with a bleeding ulcer **(QoE: moderate)**.

#### Recommendation/4

In the high suspicion and diagnosis of staple line leak, gastric fistula, delayed gastro-jejunal anastomotic leakage, marginal or stomal ulceration, gastro-gastric fistula, gastro-jejunal anastomotic stricture and intra-luminal bleeding after LSG and LRYGB, we recommend an urgent endoscopic assessment and management in hemodynamically stable patients, according to the availability of an endoscopist with expertise in bariatric surgery. We recommend against delaying surgical exploration in hemodynamically unstable patients **(Strong recommendation based on low level of evidence 1C)**.

#### Discussion of evidence

With the development and improvement of several techniques in the management of post-bariatric surgery complications, endoscopy has gained an important role as primary tool in the assessment and management of those complex patients by expert teams.

Among all complications following LSG and LRYGB, fistulas and leaks are the most important.

Post-LSG leak can lead to the development of gastric fistula over time. Fistulas after LSG occur in 0.2% to 2.5% of cases and are most commonly located at the proximal third of the gastroplasty [57, 58]. Leaks are also major complications of LRYGB, occurring in 0.7% to 5% of patients. They are usually located at the gastro-jejunal anastomosis, but have also been noted at the distal esophagus, gastric pouch, remnant stomach, blind jejunal limb, and jejuno-jejunal anastomosis.

Early leaks, occurring in the first 14 days after bariatric procedure, have a higher rate of longstanding healing compared to late leaks, that occur over 4–6 weeks after surgery, and fistulae. Prolonged leaks with peri-anastomotic collection may evolve into a chronic fistula [59, 60].

In unstable patients in case of diffuse peritonitis, surgical exploration with peritoneal irrigation and drainage of any collection is required. The endoscopic management has been shown to be an effective and less invasive approach for stable patients.

Several endoscopic devices and techniques are available, including self-expanding metal (SEMS) and plastic stents, clipping techniques [including the use of through-the-scope clips (TTSC) and over-the-scope clips (OTSC)], tissue sealants, suturing systems (OverStitch System®), internal drainage techniques, and vacuum therapy. These techniques may be applied alone or in combination, and as first-line or as salvage treatment after failure of previous approaches. Unfortunately, a standardized approach that fits for all possible scenarios does not exist. The choice of one procedure over another depends on the clinical presentation, defect features, and local expertise [61].

Adequate knowledge of postsurgical gastrointestinal anatomy and close collaboration with the bariatric surgeon and radiologist are advised to reduce procedure related complications and improve outcomes**.** Moreover, insufflation of CO2 is recommended to reduce the risk of barotraumas on surgical suture, in early postoperative endoscopy, and pneumoperitoneum or pneumomediastinum, in cases of suspected leak [62, 63].

Deployment of fully covered self-expanding metal stents (FC-SEMS) is the most common technique used to treat fistulae [64, 65]. The rationale of stent deployment is to seal the defect and divert luminal fluid, allowing the gastrointestinal wall to heal. Unfortunately, the use of FC-SEMS is burdened with a high rate of adverse events [66]. Furthermore, a recent study comparing patients undergoing stent placement to those requiring re-operation showed that nearly 40% of patients who had stent placement as a part of their treatment required additional interventions with additional costs and risks [66].

Specifically designed SEMS have been developed for the management of leaks after bariatric surgery. These stents were designed to reduce the risk of migration and to adapt well to the post-bariatric surgery anatomy (mainly LSG). Nonetheless, they had similar success rate, and migration rate compared with “standard SEMS” [67].

The endoscopic internal drainage (EID) includes the deployment of one or more plastic double pigtail stents through the leak orifice in order to internally drain any extra-luminal fluid collection and promote secondary healing. EID has recently emerged as an effective option in the endoscopic management of leaks. Moreover, plastic stents acting as a foreign body, induce mechanical trauma promoting cavity re-epithelialization [68]. Although multiple endoscopic sessions are required to treat leaks, EID success rate ranges from 78 to 86% [69, 70]. This technique is replacing the “stent strategy” in the management of bariatric leak due to its high success rate, lower complications rate and better cost-effectiveness [71–73].

The intra-cavitary endoscopic vacuum therapy (EVT) with endo-sponge (B Braun Medical BV, Melsungen, Germany) is an endoscopic technique that allows optimal drainage of the cavity, ensuring granulation, according to the concept of keeping the leak open. In contrast, EID guides drainage toward the GI tract, obstructs the leak orifice, and enables oral intake while favoring mechanical re-epithelialization. One of the great disadvantages of EVT is the need for repeated endoscopic procedures because the sponge needs to be changed every 3 to 5 days and leak closure might be more difficult to achieve with intra-luminal EVT alone. EID can be used in acute and chronic leaks with associated collections; better results may be achieved with intra-abdominal leaks and when several pigtail stents can be delivered side by side in an attempt to occlude the leak defect. Some see no value in routine stent exchange in EID unless necrosectomy is also performed [74, 75].

Endoscopic septotomy is a novel approach used to facilitate internal drainage of refractory leaks and fistulae by incision and enlargement of the fistulous tract (septum) to equalize the pressures between the gastric lumen and peri-gastric collection similarly to endoscopic Zenker’s diverticulotomy. Small case series have shown efficacy of septotomy, particularly in chronic settings [76, 77].

Direct leak closure with endoscopic suturing system has been recently proposed in small series of patients with good outcomes [78, 79]. However, further studies are needed to confirm the long-term efficacy of this approach.

Postoperative stricture is one of the most common late complications after bariatric surgery.

After LRYGB, strictures occur mainly at the gastro-jejunal anastomosis with an incidence ranging from 3 to 12% [80, 81]. Less frequently strictures occur at the entero-enteric anastomosis or, in case of retrocolic approach, at the passage of the Roux limb across the mesocolon [82].

Through-the-scope (TTS) balloon dilatation achieves stricture resolution in more than 90% of cases [83]. Gradual dilation over multiple endoscopic sessions (every 2–3 weeks) is advised to reduce the rate of perforation [84]. SEMS placement has been described in small case series with suboptimal results because of the major risk of migration and secondary small-bowel obstruction [85].

The incidence of strictures after LSG ranges from 0.1 to 3.9% [86]. Post-SG strictures occur most frequently at the incisura angularis or more proximal at the gastroesophageal junction and are related mainly to luminal narrowing or torsional scarring (functional stenosis) from improper alignment of the staple line along the greater curvature [87, 88].

Management of LSG stricture is more challenging than LRYGB stomal strictures because it requires a larger dilation. TTS balloon dilation treatment is often suboptimal. The treatment of choice is dilation with the achalasia balloon (30–35–40 mm) due to its larger diameter and rigidity, becoming more effective in tearing the fibrotic tissue of the stenotic area [89]. In a recent meta-analysis including 426 patients, Chang et al. [90] concluded that endoscopic dilatation is a minimally invasive approach that should be proposed as first-line treatment. It has an overall success rate of 76% with a rate of adverse events of 0.9% and 17% of cases require revisional surgery for definite treatment.

Early postoperative bleeding is more frequent observed after LRYGB with an incidence ranging from 1 to 4% [91] compared to less than 2% after LSG [92]. Early postoperative bleeding may be intra-luminal or extra-luminal (half of the cases) and occurs most frequently at the staple line or at the anastomosis (++ gastro-jejunal and +/‒ jejuno-jejunal).

Endoscopic evaluation should be performed by an expert endoscopist with CO2 insufflation due to higher risk of perforation and dehiscence at the anastomotic site. Injection and mechanical techniques should be preferred over thermal technique in order to minimize the risk of ischemia and anastomotic necrosis [89]. Injection of tissue adhesive has been reported in small case series showing a high success rate and safety profile. Recently hemostatic powders have been used allowing the treatment of large bleeding areas [93, 94]

Bleeding can occur lately after LRYGB, and it is mostly related to marginal ulcer (ulceration located on the jejunal side of the anastomosis) or stomal ulcer (ulceration on the gastric side).

Local ischemia is the main cause of stomal ulcer, whereas exposure to acid contents seems to play an important role for marginal ulcers. The endoscopic approach is advised in stable patients in case of acute massive bleeding or in case of bleeding relapse after medical treatment with proton pump inhibitors or antacids. Endoscopic techniques for management of post-LRYGB ulcers do not differ from the standard approaches for peptic ulcers [95, 96].

Close collaboration between surgeons, interventional radiologists, and therapeutic endoscopists is recommended to optimize outcomes of long-term complications after bariatric surgery.

### Q.5: HOW SHOULD ANTIBIOTICS BE USED IN THE MANAGEMENT OF THE ACUTE ABDOMEN IN PATIENTS WITH PREVIOUS HISTORY OF BARIATRIC SURGERY?

#### Statement 5.1

The optimal management of patients presenting with sepsis requires early source control, adequate empiric antimicrobial therapy, and targeted fluid resuscitation **(QoE: moderate)**.

#### Statement 5.2

Complicated IAIs are multi-bacterial mainly caused by Gram-negative bacilli and anaerobes. Broad-spectrum single-agent or combination drug regimens targeting these microorganisms are the mainstay of early empiric antimicrobial therapy **(QoE: moderate)**.

#### Statement 5.3

Obesity alone is not associated with antimicrobial treatment failure among patients with IAI. Obesity may not be an absolute indication for longer duration of antimicrobial therapy in treatment of IAI **(QoE: moderate)**.

#### Statement 5.4

A short (3–4 days of IV antibiotics) course of therapy may be effective even in critically ill surgical patients with complicated IAI, including fungal infection, after adequate source control **(QoE: moderate)**.

#### Statement 5.5

The empiric implementation of antifungal therapy due to perforated marginal ulcer (ulceration located on the jejunal side of the anastomosis) or stomal ulcer (ulceration on the gastric side) is not supported by the literature in community-acquired IAI **(QoE: moderate)**.

#### Statement 5.6

The dose of antibiotics must be adjusted to the weight and renal function of the patient** (QoE: high)**.

#### Recommendations/5

We recommend administering early empiric broad-spectrum antimicrobial therapy in patients presenting with intra-abdominal infections, in addiction to adequate source control. After surgical management, short-term antimicrobial therapy is preferred even in critically ill patients **(Strong recommendation based on moderate quality of evidence 1B)**.

We suggest administering antifungal therapy in frail, immunocompromised patients, presenting with biological and clinical signs of sepsis, and if fungal organisms are isolated in the intraperitoneal fluid **(Weak recommendation based on low quality of evidence 2C)**.

#### Discussion of evidence

Severe intra-abdominal infections (IAI) could complicate bariatric procedures, due to peritonitis or intra-abdominal abscesses formation. In addition, cases of IAI are often classified as uncomplicated or complicated. “Complicated” describes extension of infection from their source into the peritoneal cavity.

An easier system classifies intra-abdominal infections according to their acquisition setting in community-acquired, healthcare-associated or early onset hospital-acquired, or late-onset hospital-acquired; the presence of anatomical disruption (either absent or present resulting in localized or diffuse peritonitis), and severity of disease expression (infection, sepsis, or septic shock). This classification defines different phenotypes of the same disease by covering aspects of (i) the extent of intra-abdominal contamination reflecting the complexity of source control, (ii) the level of associated organ disfunction/failure indicating sense of urgency and prognosis, and (iii) likelihood of the presence of antimicrobial resistant microorganisms or otherwise important pathogens which may require broader antimicrobial coverage (enterococci, *Candida* spp.) [97].

The optimal management of patients presenting with signs of shock requires aggressive fluid resuscitation, adequate empiric antimicrobial treatment, and early source control.

Complicated IAIs are caused by a wide variety of microorganisms, including both aerobes and anaerobes, Gram + and Gram-. Enterobacteriaceae, in combination with anaerobes, are the most common microorganisms observed in community-acquired complicated IAIs, whereas other microorganisms such as Pseudomonas aeruginosa, Staphylococcus aureus, Enterococcus spp., and Candida spp. can play a crucial role in healthcare-acquired complicated IAIs.

In a French multicenter study, Montravers et al. found increased numbers of E. faecalis and P. aeruginosa isolates in healthcare-acquired complicated IAIs compared to community-acquired IAIs (33% vs. 19% [*P* < 0.05] and 13% vs. 5% [*P* < 0.01], respectively), whereas in community-acquired complicated IAIs, the most commonly isolated microorganisms were Escherichia coli, Streptococcus, and Bacteroides fragilis [98].

A complicated IAI in a patient with a previous history of bariatric surgery should be considered a healthcare-acquired infection rather than a community-acquired infection, because of the recent hospitalization, antibiotics use and immunosuppression due to malnutrition and sepsis. Intra-operative cultures and blood cultures should be carried out, to eliminate also the co-presence of multi-drug resistant bacteria.

In recent years, an increasing number of community-acquired complicated IAIs have been caused by ESBL-producing *Enterobacteriaceae,* with a higher rate of inadequate antimicrobial treatment, which is associated with higher mortality rate, longer hospital stay, and increased cost compared to adequate antimicrobial treatment [99].

Initial antibiotic therapy should be empirical and be adjusted to microbiological data (culture and susceptibility results) [100].

Evidence (single-center, retrospective) suggests that a short (3–4 days of IV antibiotics) course of therapy may be effective even in critically ill surgical patients with abdominal sepsis after adequate source control, according to clinical and biological parameters re-evaluations [101].

The STOP-IT study showed that obesity is not associated with antimicrobial treatment failure among patients with IAIs. This suggests that obesity may not independently influence the need for longer duration of antimicrobial therapy in treatment of IAI compared to non-obese patients [102].

The empiric implementation of antifungal therapy in marginal ulcer (ulceration located on the jejunal side of the anastomosis) or stomal ulcer (ulceration on the gastric side) is not supported by the literature in a community-acquired IAI.

The current evidence (one randomized multicenter trial evaluating outcomes for patients with intra-abdominal perforations, including perforated peptic ulcer; one single-center prospective series and three retrospective studies) comparing outcomes in patients with perforated peptic ulcer (PPU) treated with or without empiric antifungal therapy did not showed efficacy of antifungal agents in improving outcomes. Therefore, the routine empiric use of antifungal therapy in non-critically ill or non-immunocompromised patients presenting with gastrointestinal perforation should not be routinely recommended [103].

Fungal isolates from peritoneal fluid sampling in patients with PPU are not uncommon.

A retrospective study including 133 patients suffering community-acquired PPU-associated peritonitis with Candida species isolated from their peritoneal fluid showed that antifungal therapies do not improve outcomes, but it could be reserved for patients who are critically ill and/or severely immunocompromised [104].

Kwan et al. in a single-center retrospective study including adult patients with perforated gastric and duodenal ulcers over a 10-year period showed that old age (median age, 64; IQR 53–74) is a predictor of fungal growth (*p* < 0.001) but that fungal growth is not a predictor of adverse perioperative outcomes [105].

In addition, critically ill patients with IAI involving fungal organisms randomized to a shorter course of antimicrobial therapy had no difference in the rate of treatment failure. These results suggest that the presence of fungi in IAI may not independently indicate the need for longer course of antimicrobial therapy [106].

### Q.6: HOW CAN WE DECREASE THE RISK OF THROMBOEMBOLIC EVENTS IN SURGICAL PATIENTS WITH A PREVIOUS HISTORY OF BARIATRIC PROCEDURES AFTER EMERGENCY SURGERY?

#### Statement 6.1

All patients needing an urgent surgical procedure should be risk stratified for venous thromboembolism **(QoE: moderate)**.

#### Statement 6.2

In the emergency setting, venous thromboembolism pharmacologic prophylaxis should be started as soon as possible, if there are no signs of active bleeding, to decrease the risk of venous thromboembolism **(QoE: moderate)**.

#### Statement 6.3

If contraindications to pharmacologic venous thromboembolism prophylaxis exist, mechanical venous thromboembolism prevention strategies such as compression stockings and foot pump should be considered even if the patient is ambulating, because of the high venous thromboembolism risk following emergency surgery **(QoE: moderate)**.

#### Statement 6.4

Pharmacologic venous thromboembolism prophylaxis with unfractionated heparin or low-molecular-weight heparin is highly effective for the prevention of venous thromboembolism in hospitalized patients **(QoE: moderate)**.

#### Statement 6.5

The venous thromboembolism prophylaxis should continue after discharge according to the thrombotic risk. This can be estimated based on patient- and procedure-specific factors, applying validated risk assessment models such as the Caprini score. Patients with a prolonged hospital stay, the presence of cancer, urinary tract infection, and postoperative sepsis are at high risk to present with venous thromboembolism **(QoE: moderate)**.

#### Recommendation/6

We recommend the administration of low-molecular-weight heparin for venous thromboembolism prophylaxis as soon as possible in patients presenting with an acute surgical abdomen after bariatric surgery. The LMWH dose should be adjusted to the patient’s weight, the thrombotic risk and creatinine clearance **(Strong recommendation based on moderate level evidence 1B)**.

We suggest to continue venous thromboembolism prophylaxis at least 4 weeks after discharge **(Weak recommendation based on moderate level evidence 2B)**.

We suggest to use the monitoring of anti-Xa levels to adjust LMWH dose, particularly in elderly, pregnant, renally impaired, with BMI =  > 35 kg/m^2^ patients, at risk of suboptimal dosing **(Weak recommendation based on moderate level evidence 2B)**.

In patients where pharmacologic venous thromboembolism prophylaxis is contraindicated, we recommend the use of mechanical prophylaxis, especially in high-risk patients **(Strong recommendation based on moderate level evidence 1B)**.

#### Discussion of evidence

Venous thromboembolism (VTE) is a frequent complication in surgical patients which contributes to increased morbidity and mortality. Deep vein thrombosis (DVT) occurs in 1–3%, and a pulmonary embolism (PE) in 0.3–2% of patients during and after bariatric surgery [107, 108]. The reported rate of VTE among patients undergoing emergency surgery is approximately 2.5% [109].

Particularly in patients with obesity, the increased risk of VTE is not only related to clinical factors such as venous stasis or immobility, but also due to a chronic state of inflammation of the adipose tissue that causes alterations in the hemostatic process. Therefore, obesity and high BMI are independent risk factors for DVT and PE [110] in patients needing urgent surgical procedures in addition to patient-specific factors (e.g., a history of VTE).

Finks et al. [111] have identified the risk factors related to the incidence of VTE in patients after bariatric surgery including sex (male), age (> 60 years), smoking, and high BMI. In addition, compared with elective surgical patients, emergency surgical patients are at an increased risk of VTE.

Ross et al. [112] used the American College of Surgeons National Surgical Quality Improvement Program database to carry out a retrospective cohort study on 604 537 adults undergoing surgical procedures over 12 years to investigate whether emergency surgery is independently associated with VTE compared with elective surgery. They found that emergency surgery, open surgery and partial colectomy were independently associated with VTE.

At the initial evaluation of the patient, it is important to assess the risk for VTE.

The Caprini assessment tool is the most used in clinical practice. It considers individual risk factors such as age, weight, personal history of VTE, and surgery-related variables such as the type and the length of the procedure. The Caprini risk model divides patients into three categories based on the VTE risk:

-*Low-risk patients* (with a score of less than five) do not need postoperatively anticoagulant prophylaxis since the incidence of 30-day clinically evident venous thromboembolism events is less or equal to the risk of bleeding from anticoagulation.
-*Standard-risk patients* (with a score between five and eight) are those with a risk score reflecting a VTE incidence that exceeds the incidence of bleeding events using anticoagulation.
-*High-risk patients* (score > 8) are those that are at great risk of developing venous thromboembolism postoperatively, and merit anticoagulant protection for 30 days [113].

Strategies to reduce the incidence of VTE include: mechanical (graduated compression stockings, intermittent pneumatic compression, sequential compression devices, foot pumps, early ambulation) or pharmacologic (low-molecular-weight heparin [LMWH] and unfractionated heparin [UFH]).

Unlike pharmacologic prophylaxis, mechanical prophylaxis is not associated with an increased risk of bleeding complications. Furthermore, there are few contraindications to mechanical prophylaxis, including lower-extremity wounds and severe peripheral arterial disease.

Pharmacologic VTE prophylaxis is highly effective for VTE prevention in hospitalized patients.

Bergqvist et al. [114] reported a reduction of postoperative VTE from 22 to 8% with the use of LMWH in emergency surgery patients.

Contraindications to pharmacologic prophylaxis include active bleeding or the presence of disorders associated with a significant risk of bleeding, such as thrombocytopenia or coagulopathy.

DeWane et al. [115] retrospectively analyzed 130,036 patients in the American College of Surgeons NSQIP database who underwent emergency surgery and reported that more than 30% of VTEs occur after discharge and that the majority of these patients required readmission. Predictive factors for post-discharge VTE included prolonged length of stay (odds ratio [OR] 5.25; *p* < 0.001), the presence of metastatic cancer (OR 2.23; *p* < 0.001), urinary tract infection (OR 1.91; *p* < 0.001), and postoperative sepsis (OR 1.55; *p* < 0.001).

A recent systematic review of the literature showed that using a validated system for risk assessment and prescribing LMWH for all operative and non-operative abdominal emergencies, and using mechanical prophylaxis if pharmacologic prophylaxis is contraindicated may improve the management of patients requiring an urgent surgical procedure for intra-abdominal surgical diseases [116].

Currently there are no recommendations concerning the clinical relevance of routine anti-Xa levels monitoring for LMWH dose adjustment in patients on VTE thromboembolism prophylaxis, except in elderly, pregnant, renally impaired, with BMI > 35 kg/m2 patients, being at risk of suboptimal dosing [117, 118].

In patients for whom LMWH monitoring is required, anti-Xa levels should be obtained 4 h after administration. A reasonable anti-Xa target range for LMWH DVT prophylaxis is suggested to be 0.2–0.5 IU/mL [117, 118].

### Q.7: WHICH SURGICAL PROCEDURES ARE EFFECTIVE IN THE MANAGEMENT OF ACUTE ABDOMEN FOLLOWING BARIATRIC SURGERY?

**Scenario 1: BLEEDING**

#### Statement 7.1

Intra-abdominal bleeding occurs very rarely as a late complication after bariatric surgery **(QoE: low)**.

#### Statement 7.2

The most common reason for late gastrointestinal bleeding is ulcer from the gastric sleeve and gastro-jejunostomy, gastric pouch, bypassed stomach, or duodenum after LRYGB **(QoE: moderate)**.

#### Statement 7.3

Bleeding after LRYGB and LSG is self-limiting in many patients and can be managed conservatively, with medical treatment and close monitoring and observation **(QoE: low)**.

#### Statement 7.4

In hemodynamic stable patients presenting with intra-luminal intestinal bleeding after bariatric surgery, the endoscopic assessment (that must be performed after endotracheal intubation to protect airways) is a valid and safe tool after bariatric surgery **(QoE: moderate)**.

#### Statement 7.5

Angiography and angioembolization may be valid strategies to bleeding control **(QoE: very low)**.

#### Statement 7.6

If endoscopic and angiographic management fail, surgical exploration is indicated, if there are signs of persisting bleeding **(QoE: low)**.

#### Statement 7.7

In unstable patients not responding to aggressive resuscitation, diagnostic laparotomy and surgical hemostasis are mandatory **(QoE: high)**.

#### Recommendations/7-BLEEDING

Endoscopy is the first recommended diagnostic tool in stable patients presenting with gastrointestinal bleeding after SG and RYGB **(Strong recommendation based on moderate quality evidence 1B)**.

We suggest performing an angio-CT and embolization in stable patients presenting with gastrointestinal and intraperitoneal extra-luminal bleeding, when skills are available **(Weak recommendation based on low level of evidence 2C)**.

In selected cases of hemodynamically stable bleeding peptic ulcer patients, after failure to attempt endoscopic hemostasis, we suggest the use of angiography with angioembolization if technical skills and equipment are available **(Weak recommendation based on very low level of evidence 2D)**.

We recommend against delaying surgical exploration in unstable patients presenting with ongoing gastrointestinal bleeding after endoscopic assessment and negative angio-CT scan for bleeding source localization **(Strong recommendation based on low level evidence 1C)**.

In patients selected for surgical exploration for a bleeding ulcer, we suggest planning intraoperative endoscopy to facilitate the localization of the bleeding site, using a surgical gastrostomy in case of patient with LRYGB to assess the gastric remnant and duodenum **(Weak recommendation based on very low level of evidence 2D)**.

We recommend against delaying diagnostic laparoscopy/laparotomy in patients presenting with ongoing intraperitoneal extra-luminal bleeding, after angioembolization **(Strong recommendation based on low level of evidence 1C)**.

A biopsy of the bleeding ulcer is recommended to exclude malignancy **(Strong recommendation based on low level of evidence 1C)**.

#### SUMMARY AND DISCUSSION OF EVIDENCE

Gastrointestinal bleeding after LSG is a rare late complication which is caused essentially by a gastric ulcer.

Several studies [119–121] have associated LSG with gastroesophageal reflux causing gastric ulceration and bleeding.

Bleeding gastric ulcers after LSG can be treated similar to a bleeding upper gastrointestinal ulcer occurring in the general population. According to the WSES guidelines [122], if the patient is hemodynamically stable, an early endoscopic evaluation of the stomach is recommended to establish the diagnosis and to treat the bleeding. Surgical hemostasis is recommended in patients with recurrent bleeding from an ulcer greater than 2 cm, with intra-operative endoscopic assessment of the source of bleeding.

After LRYGB, the marginal ulcer is the most common complication. The incidence of marginal ulcers (MU) ranges from 0.6 to 16%. It can lead to bleeding and perforation and may require surgical urgent treatment. It has been estimated that 1% of LRYGB patients will suffer from a perforated marginal ulcer in their life. Patients with a history of smoking, immunosuppression and preoperative non-steroidal anti-inflammatory drugs use were significantly more likely to develop a MU requiring surgical revision. Patients undergoing revision had a resolution of their symptoms in only 36% of the cases, while 57% developed a recurrent ulcer [123].

Marginal ulcers are classified into early and late ulcers depending on the timing of the diagnosis.

The development of *an early* marginal ulcer, which occurs 1 to 10 months after surgery, is more likely to be associated with local factors (ischemia, postoperative inflammation, stenosis, and the presence of a foreign body), while *late* marginal ulcers are likely related to increased acid exposure of the gastro-jejunal anastomosis developing over time [124]. For both types of ulcers, when the diagnosis is made, the treatment is medical and consists of a minimum of 3 to 6 months of proton pump inhibitors therapy, elimination of potential risks factors, and regular endoscopic control to monitor healing and rule out stenosis. [125].

Recurrent marginal ulcers refractory to medical therapy are often due to local problems such as enlargement of the gastric pouch over time or presence of a gastro-gastric fistula with subsequent increased acid exposure of the jejunal mucosa. The presence of a Zollinger–Ellison syndrome has to be ruled out in these patients. It was reported that refractory marginal ulcers after gastric bypass demand revisional surgery. The operation consists of resection and reconstruction of the gastro-jejunal anastomosis with or without partial remnant gastrectomy.

The management of marginal ulcers complicated with bleeding, in the emergency setting, should follow the WSES guidelines: In hemodynamically stable patients, the first assessment should be by endoscopy, which may achieve hemostasis and reduce re-bleeding, the need for surgery, and mortality [122]. Angiography with transcatheter angioembolization could be a therapeutic option in case of failure of endoscopic hemostasis or unavailability of the procedure. In patients with refractory bleeding peptic ulcer, surgical intervention is mandatory with intra-operative endoscopy to facilitate the localization of the bleeding. An immediate or delayed biopsy is always recommended [122].

The management of bleeding from excluded segments of post-RYGB, such as gastric remnant and duodenum, is more challenging. Endoscopy is considered the first-line approach for the diagnosis and treatment of upper gastrointestinal bleeding, but the access is limited due to the altered anatomy of the gastric remnant and duodenum. In the presence of an experienced endoscopists, the gastric remnant and duodenum can be reached with a pediatric colonoscope in up to 68% of cases [126]. This rate can be raised to 88% by applying a double-balloon technique although it has a perforation rate of 10% [127].

In emergency situations, the endoscopic assessment of the duodenum and gastric remnant can be achieved by performing a temporary laparoscopic surgical gastrostomy through the gastric remnant allowing to perform a transgastric endoscopy assessment [128]. In any case, a biopsy of the ulcer is always recommended to exclude the presence of a neoplasm.

### Scenario 2: INTESTINAL OBSTRUCTION

#### Statement 7.8

Stenosis after LSG in stable patients presenting with gastrointestinal symptoms should be assessed by endoscopy, and the treatment must be tailored according to the clinical status of the patient and endoscopic findings **(QoE: low)**.

#### Statement 7.9

Endoscopic pneumatic dilation is a safe and effective first-line treatment of gastric stenosis after SG and gastro-jejunostomy strictures after RYGB. Perforation is a potential complication of this treatment and may necessitate surgical intervention **(QoE: moderate)**.

#### Statement 7.10

After RYGB, the most common causes of small-bowel obstruction (SBO) are internal hernia, adhesions, incisional hernia/trocar site hernia, intussusception, volvulus, obstruction localized at the jejuno-jejunostomy (JJ), twisted alimentary limb, alimentary limb mesenteric ischemia, and adhesions proximal to the JJ **(QoE: low)**.

#### Statement 7.11

In case of SBO after RYGB, an exploratory laparoscopy is mandatory in the first 12–24 h in stable patients presenting with persistent abdominal pain and inconclusive clinical and radiological findings **(QoE: low)**.

#### Statement 7.12

Diagnostic laparoscopy in pregnant women with SBO after bariatric surgery is effective and is associated with a good maternal and fetal outcome **(QoE: very low)**.

#### Statement 7.13

Surgical exploration in patients after LRYGB should start from the ileocecal junction (distally to the obstruction) toward the inspection of the jejuno-jejunostomy and of the 3 potential site locations of internal hernia: the traverse mesocolon (in retrocolic bypasses), Petersen’s space (Petersen’s hernia) and the jejuno-jejunostomy mesenteric defect (mesojejunal hernia), and then the remnant stomach. **(QoE: very low)**.

#### Statement 7.14

If an internal hernia is found, an assessment of intestinal viability should be undertaken; if intestinal ischemia is present, surgical resection is performed. The closure of the mesenteric defect should be performed with non-absorbable material in running or interrupted suture **(QoE: very low)**.

#### Statement 7.15

Indocyanine green (ICG) fluorescence angiography may be a valid tool in the evaluation of the extent of bowel resection and anastomosis perfusion, when it is available **(QoE: very low)**.

#### Statement 7.16

If no internal hernia or other evident causes of SBO are found, the entire small intestine should be assessed given that there are other causes of intestinal obstruction (adhesions, intussusception, volvulus) **(QoE: very low)**.

#### Statement 7.17

In case of intra-operative findings of intussusception, the surgical treatment may be limited to reduction if the small bowel is viable, but resection of the affected segment is recommended since it seems to result in fewer recurrences **(QoE: very low)**.

#### Statement 7.18

In the emergency setting, when SBO is due to a bezoar located in the stomach, the first approach could be endoscopy. If the bezoar is located distally in the small bowel, surgical intervention is required to milk the bezoar into the cecum or remove it by creating an enterotomy **(QoE: very low)**.

#### Recommendations/7-INTESTINAL OBSTRUCTION

In the presence of symptoms of proximal SBO after LSG and LRYGB, endoscopic assessment is recommended in stable patients **(Strong recommendation based on low level of evidence 1C)**.

In the presence of SBO after RYGB, we recommend performing an exploratory laparoscopy in the first 12–24 h in stable patients with a history of bariatric surgery presenting with persisting acute abdominal pain after inconclusive laboratory, radiological and endoscopic results in the emergency setting (**Strong recommendation based on low level of evidence 1C)**.

We suggest considering limited intestinal resection and anastomosis in case of clear intestinal segmental ischemia in hemodynamically stable patients, or damage control and open abdomen approach in cases of extended intestinal ischemia/peritonitis in hemodynamic unstable patients **(Weak recommendation based on low level evidence 2C)**.

#### Summary and discussion of evidence

Gastric obstruction after SG occurs in 0.2–4% of cases and most cases of obstruction present within 6 weeks after surgery [129]. It can be caused by a mechanical narrowing, usually located at the incisura angularis or an axial obstruction due to rotation phenomenon secondary to incongruence between the anterior and posterior gastric wall [130, 131]. Most of stenoses are located in the proximal or distal third of SG with the incisura angularis reported to be the most prevalent location for obstruction [130, 131]. Predisposing factors for gastric strictures after SG are calibrating the stomach with smaller bougie diameter, stapling too close to the incisura angularis, postoperative edema, or hematoma [129].

The endoscopic management of these strictures with balloon dilatation or stent placement, is reported to be successful in 88–94% of cases [132–134]. When endoscopic methods are unsuccessful, SG conversion to RYGB should be considered [129].

In the emergency setting, patients presenting with symptoms of obstruction such as nausea, vomiting, and intolerance to solid food intake after SG may benefit from a naso-gastric tube placement to decompress the stomach, before assessing the SG by endoscopy.

After RYGB, the overall incidence of SBO ranges from 6 to 9.6%, and it could occur late (more than 30 days after surgery), usually resulting from an internal hernia or adhesions, or early (within 30 days of surgery) which is more commonly attributed to technical problems with the Roux limb, such as kinking or obstruction at the jejuno-jejunostomy [135, 136].

After RYGB, patients rarely present with vomiting because of the small size of the gastric pouch. If bilious vomiting is present, it is a sign that the origin of the obstruction is at the jejuno-jejunostomy. More rarely, SBO could be due to a gastro-gastric fistula which allows the passage of the bile from the remnant stomach to the gastric pouch [135].

The most common causes of SBO after RYGB are internal hernia, adhesive disease, jejuno-jejunostomy stenosis or kinking, incisional hernia, intussusception, and bezoar [136].

Husain et al. [137] retrospectively reviewed 2325 patients. The most common causes of late SBO after RYGBP were internal hernia (53.9%), Roux limb compression due to mesocolon scarring as it passed through the mesocolic window (20.5%), and adhesions (13.7%). Laparoscopic explorations were carried out in 92 cases (82.9%).

The incidence of SBO caused by internal hernia is higher with retrocolic Roux placement when compared with antecolic Roux placement. In patients with antecolic Roux placement, the most common cause of obstruction is adhesive bands or stenosis at entero-enterostomy [137].

When suspecting an internal hernia, in hemodynamically stable patients, early explorative laparoscopy is mandatory to avoid late diagnosis, intestinal vascular compromise, and bowel resection.

It is suggested to begin the exploration from the alimentary limb at the gastro-jejunal anastomosis. This limb must be followed distally to its junction with the transverse colon where Petersen’s space will be evaluated. The exploration should be carried on through the alimentary limb to the jejuno-jejunal anastomosis to assess the inter-mesenteric defect.

Internal hernia can be easily reduced from the ileocecal valve, distal to the obstruction, where the intestine is less dilated and can be handled much safer by laparoscopy. After reducing the hernia, if the bowel loops are viable, all the mesenteric defects and Petersen’s defect have to be closed with non-absorbable sutures.

If an intestinal resection is required because of vascular compromise, indocyanine green (ICG) fluorescence angiography can support surgical decision making in evaluating intestinal perfusion and may help to define resection margins more accurately [138].

If no internal hernia is found, the entire small intestine should be assessed given that there are other causes of SBO (adhesions, intussusception, volvulus) that should be discarded in these patients. [139].

Another cause of obstruction after RYGB is stenosis at the GJ. It has a reported incidence ranging between 3 and 27% [140]. Risk factors for developing GJ stricture are: the size of circular staple used in confectioning anastomosis, the retrocolic or antecolic positioning of the Roux limb, and the initial size of the anastomosis [141]. The presence of a gastro-gastric fistula can result in an anastomotic stricture due to the large amount of acid (bile) that flows from the gastric remnant into the pouch responsible of a chronic marginal ulcer [142].

GJ strictures can be classified based on the mechanism of formation and endoscopic evaluation. These classifications can guide decision making. For example, membranous strictures are easily treated by endoscopic hydrostatic balloon dilatation; cicatricial strictures are characterized by intense fibrosis and respond unpredictably to endoscopic balloon dilation; granular strictures, that seem to result from secondary intention healing, or from tissue necrosis, require an early endoscopic approach [143].

If the endoscopic treatment fails, surgical revision is an option to consider with an expert bariatric surgeon. Laparoscopic revision of a strictured anastomosis is a technically challenging procedure because of adhesion formation and difficulties in anatomical identification, even if an antecolic antegastric route of the Roux limb may make this attempt easier.

In the emergency setting, SBO due to GJ stenosis can be managed by decompressing the gastric pouch with the placement of a naso-gastric tube and an endoscopic assessment to provide diagnosis and treatment.

SBO after LRYGB could be due to intussusception, with an incidence estimated range of 0.1% to 0.3% [144]. The pathophysiologic mechanism of intussusception is complex and involves modified intestinal motility, the presence of staple lines and other lead points in the intestinal wall, and anatomic peculiarities at entero-enterostomy [145].

Intussusception can be classified in antegrade when the lead point is usually identifiable and can involve either limb; and retrograde (anti-peristaltic), which is the most common form after LRYGB. It is characterized by a featureless entry point beginning a few inches below the intestinal anastomosis, with the intussusceptum traversing the entero-enterostomy into either the biliary or Roux limb [146].

The anatomical classification for jejunogastric intussusceptions which is widely accepted was proposed by Schackman et al. [147] which categorized the jejunogastric intussusception into 3 types:

- Type I—Afferent loop intussusception (antegrade);
- Type II—Efferent loop intussusception (retrograde);
- Type III—combined form of intussusception.

The management of jejunogastric intussusception could be endoscopic in selected cases, considering that the endoscopic reduction of intussusception is associated with an increased rate of recurrence [160]. Surgical exploration in laparoscopy or by laparotomy, according to the hemodynamic status of the patient, is recommended because of the high risk of incarceration and strangulation. Delay in surgical intervention is associated with a significant increase in mortality especially after 48 h [148].

However, there is no consensus about the best surgical treatment of intussusception after LRYGB. Gentle manual reduction of intussusception could be possible with high risk of recurrence. If there is intestinal necrosis, the nonviable segment should be resected with the creation of a new anastomosis when it is allowed (patients without signs of hemodynamic instability). If the involved segment includes the jejuno-jejunostomy, the latter will need reconstruction. Other described surgical techniques include anchoring of efferent limb to surrounding structures like parietal peritoneum and the Noble enteropexy. Reversal of the gastric bypass and conversion to another procedure like sleeve gastrectomy may be an option, but it has to be evaluated by an experienced bariatric surgeon in a multidisciplinary approach [149].

To our knowledge, resection of the invaginated segment is the treatment of choice for avoiding recurrences. Laparoscopy or open surgery can be used, depending on the experience of the surgeon and the setting.

Another rare cause of SBO after LSG or LRYGB, is the occurrence of a bezoar.

Risk factors for bezoar formation are reduced gastric motility, loss of pyloric function, hypoacidity, low mastication and high consumption of high-fiber foods. After RYGB, the relatively restricting gastro-jejunostomy and a small gastric pouch are risk factors for presenting phytobezoar [150, 151].

The management of patients presenting with SBO due to bezoar includes non-operative strategies such as chemical or endoscopic fragmentation and removal, and surgical removal, according to the site of obstruction [150, 151].

Chemical dissolution and endoscopic removal is the primary treatment of choice when the bezoar is located in the stomach. Bezoar dissolution could be obtained through oral or endoscopic injection of Coca-Cola or papain [150, 151].

Bezoars in the small bowel usually require surgical exploration and removal either by milking the bezoar into the cecum or alternatively, and less commonly, by enterotomy [150, 151].

In the emergency setting, when SBO is due to a bezoar located in the stomach, the first approach could be non-operative by endoscopy, that will allow also a balloon dilation if an anastomotic or pouch stricture is found. If the bezoar is located distally in the small bowel, surgical intervention is required to milk the bezoar in the cecum or remove it by enterotomy.

### Scenario 3: localized or generalized PERITONITIS

#### Statement 7.19

Stable patients with perforated gastro-jejunal ulcer after LRYGB should be managed with laparoscopic primary repair by suturing and omental patch which are safe and feasible and associated with decreased operative time, blood loss, and length of stay **(QoE: low)**.

#### Statement 7.20

In patients presenting with marginal ulceration and perforation at the jejuno-jejunal anastomosis, laparoscopic primary suturing is a valid option in selected patients (young patients, early presentation, no other serious comorbidities, hemodynamic stability) **(QoE: low)**.

#### Statement 7.21

If a gastro-gastric fistula is found at surgical exploration for a perforated ulcer, surgical options include simple resection of the fistula, resection of the fistula with revision of the gastro-jejunal anastomosis (GJA), resection of the fistula with remnant gastrectomy ± revision of GJA, or gastrectomy of the remnant stomach (**QoE: very low)**.

#### Statement 7.22

After LRYGB, a perforated remnant stomach may be managed by primary suture and omental patch or stapled resection, If there is concern for significant postoperative ileus due to peritonitis, a gastrostomy tube could be placed in the gastric remnant proximal to the site of the perforation to decompress the stomach and allow a postoperative endoscopic access **(QoE: low)**.

#### Statement 7.23

If diffuse peritonitis is due to a perforated excluded gastrointestinal segment (stomach or duodenum), it is recommended to explore the jejuno-jejunostomy (stenosis) or the gastric remnant (gastro-gastric fistula) **(QoE: low)**.

#### Statement 7.24

Surgical treatment of a duodenal perforation depends on the hemodynamic stability of the patient, size of the perforation and extent of native tissue loss **(QoE: very low)**.

#### Recommendations/7-PERITONITIS

We recommend performing immediate surgical exploration in unstable patients presenting with peritonitis without delay (**Strong recommendation based on low level of evidence 1C)**.

We recommend assessing all anastomoses after LRYGB, the remnant stomach and the excluded duodenum (**Strong recommendation based on low level of evidence 1C)**.

In the presence of a perforated ulcer, we recommend performing biopsies of the perforated ulceration to exclude malignancy (**Strong recommendation based on low level of evidence 1C)**.

We recommend performing primary suture with omental patch in laparoscopic approach in stable patients presenting with a perforated marginal ulcer or gastric remnant or duodenal perforation of less than1cm, whenever technically possible **(Strong recommendation based on low level of evidence 1C)**.

We suggest considering damage control surgery and open abdomen in hemodynamically unstable patients **(Weak recommendation based on low level evidence 2C)**.

#### Summary of evidence

Common causes of peritonitis in the general population may also occur after bariatric surgical procedures. We restricted our review to assess proper causes of peritonitis after SG and RYGB.

A perforated gastric or marginal ulcer should be managed according to the WSES guidelines [122]. Perforation could complicate a marginal ulcer after RYGBP with an incidence of 1% and a median time to perforation of 18 months (range 3–70 months) in large series [152]. Predisposing factors for perforated ulcer are smoking, the use of non-steroidal anti-inflammatory drugs (NSADs) and steroids [152].

A perforated gastric ulcer or marginal ulcer should be treated with laparoscopic suture repair followed by reinforcement using an omental patch. This is a safe and effective therapeutic option even in patients who have undergone bariatric surgery [153, 154].

A gastro-gastric fistula has to be suspected in the presence of a marginal perforated ulcer following gastric bypass. This has to be ruled out by exploring the gastric remnant, to decrease recurrent marginal ulcers. If a GGF is detected, it may require revision bariatric surgery by an experienced bariatric surgeon.

In the emergency setting, for a perforated ulcer it is recommended to perform laparoscopic suture repair reinforced with an omental patch. If a gastro-gastric fistula is recognized, an omental or jejunal interposition, may decrease the gastro-gastric fistula recurrence postoperatively, but only if this procedure is judged to be safe and will not extend the time of surgery.

Recently, the robotic approach in the management of bariatric surgery postoperative complications showed promising results: it was reported that it is easier to repair a perforated ulcer or an anastomotic leak assisted by robot [155, 156].

Perforation of an ulcer in the gastric remnant and in the excluded segment of the duodenum after LRYGB is rare. There are 29 cases of perforated gastric remnants reported with a perforation prevalence ranging from 0.12 to 0.84% [157]. Of those cases, 21 (72%) patients had perforation of a duodenal ulcer, seven (24%) had perforation of a gastric ulcer and one (3.4%) had simultaneous perforation of duodenal and gastric ulcers.

When the perforation is found at the gastric remnant, there is no consensus about the surgical treatment. It should be kept in mind that gastric remnant perforation could be secondary to back pressure of mechanical/functional bowel obstruction, consequently the jejuno-jejunostomy has to be assessed.

Plitzko et al. [158] reviewed the literature and found 54 cases of complicated ulcers in excluded segments after LRYGB. Out of those, 50 (93%) underwent surgery. In 48% of those patients, remnant gastrectomy was performed, combined with resection of the first portion of the duodenum in 6% and pancreas-preserving duodenal resection in 2%. Oversewing or Graham patches were performed in 37% of the patients. Other procedures included ulcer excision with bypass reversal and duodenostomy with drainage.

If a stricture or an anomaly such as kinking or twisting is found at the jejuno-jejunostomy, it has to be resected to avoid the high risk of vascular compromise and perforation.

In case of intestinal ischemia, it is recommended not to delay the surgical exploration using laparotomy if the patient is hemodynamic unstable. Laparoscopy can be used if skills are present, and the patient is hemodynamically stable. According to the extent of intestinal ischemia, principles of damage control surgery may be applied using the open abdomen by evaluating each case individually [159], although the evidence to support decision making is very limited and global cooperative randomized trials should be supported in these areas.

A very small number of cases of duodenal perforation after bariatric surgery are reported in literature. There is no consensus on the surgical management of this situation. According to the experience in the management of duodenal perforation in trauma patients [160], surgical treatment will depend on hemodynamic stability of the patient, size of the perforation and the extent of native tissue loss.

Damage control surgery should be considered in hemodynamically unstable patients. Primary repair should be considered whenever technically possible in small size duodenal perforations.

In managing large duodenal perforations (> 2 cm), there is no consensus on optimal surgical treatment. The selection of the appropriate techniques is based on the presence of an experienced surgeon, significant duodenal tissue loss, the presence of hemodynamic instability of the patient, and other operative and situational factors.

Clinch et al. [161] have recently reviewed the literature and identified 8 surgical techniques in repairing duodenal perforated ulcers: the omental plug, triple tube technique, gastric body partition, duodenojejunostomy, serial patch, pedicled patch, pancreas-sparing duodenal resection, and gastric resection.

The WSES guidelines [160] suggest using pancreas-sparing duodenectomy for ulcers in D1/D2. In perforations involving the ampulla, a definitive resectional approach is not recommended in the emergency setting because of the complexity of the reconstruction.

Damage control options, such as pyloric exclusion, gastric decompression, and external biliary drainage, should be considered contemporary to primary repair of the perforated duodenal ulcer.

### Q.8: WHAT IS THE ROLE OF DAMAGE CONTROL SURGERY IN THE MANAGEMENT OF PATIENTS WITH ACUTE ABDOMEN DUE TO LATE COMPLICATIONS OF BARIATRIC SURGERY?

#### Statement 8.1

Damage control surgery may be a tool to consider in the management of the acute abdomen in patients presenting with persistent hemodynamic instability because of severe peritonitis and septic shock **(QoE: low)**.

#### Statement 8.2

The open abdomen is an option for emergency surgery patients with severe peritonitis and severe sepsis/septic shock in the context of an abbreviated laparotomy due to severe physiological derangement, the need for a deferred intestinal anastomosis, a planned second look for intestinal ischemia, persistent source of peritonitis (failure of source control), or extensive visceral edema with concerns for the development of abdominal compartment syndrome **(QoE: low)**.

#### Recommendation/8

We suggest the use of damage control surgery with open abdomen in hemodynamic unstable patients secondary to an intra-abdominal source of infection, to extensive intestinal ischemia and massive hemoperitoneum **(Weak recommendation based on low level evidence 2C)**.

#### Discussion of evidence

Damage control surgery (DCS) represents a staged surgical approach to the treatment of critically ill patients. It has 3 stages: an abbreviated initial operative procedure with temporary abdominal closure (TAC), continued resuscitation and management of physiologic and acid–base derangements, and definitive treatment and closure [162].

At present, there are no good quality published prospective studies focused on the implementation DCS in the management of patients with a previous history of bariatric surgery. There is basic science data that open abdomen management with negative pressure peritoneal therapy may fundamentally affect the propagation of inflammatory biomediators in severe complicated intra-abdominal sepsis, although there are certainly risks to the open abdomen use. Thus, ongoing studies such as the closed or open after laparotomy for severe complicated intra-abdominal sepsis trial are attempting to answer these questions and bariatric surgery patients are not excluded from enrollment.

The threshold to operate patients in the emergency setting because of late complications of bariatric surgery, if the radiological findings are inconclusive, should be lower if they present with acute abdominal symptoms (nausea, vomiting, abdominal pain, tenderness) and/or persistent tachycardia.

Surgery is mandatory in the first 12–24 h, to obtain a good outcome and decrease morbidity and mortality rates. Laparoscopy is considered a safe technique if skills are present and if the patient is hemodynamically stable [163–165].

Acute care surgeons diagnosing surgical emergencies in patients with a previous history of bariatric surgery must not overlook the common causes of a surgical acute abdomen [108]. For these reasons, we believe that damage control surgery is a feasible option for the following conditions: hemodynamic instability, severe peritonitis and septic shock with an incomplete source control, hemorrhagic vascular injuries such as ruptured abdominal aortic aneurysm or acute mesenteric ischemia and severe acute pancreatitis, unresponsive to the step-up approach with the development of abdominal compartment syndrome [166–170].

Decompressive laparotomy is indicated in abdominal compartment syndrome if medical treatment has failed after repeated and reliable intra-abdominal pressure measurements [171].

The open abdomen is an option for emergency surgery patients with severe peritonitis and severe sepsis/septic shock under the following circumstances: abbreviated laparotomy due to severe physiological derangement, the need for a deferred intestinal anastomosis, a planned second look for intestinal ischemia, persistent source of peritonitis (failure of source control), or extensive visceral edema with concerns for the development of abdominal compartment syndrome [172].

Experience from DCS in trauma and non-trauma patients suggests that the severity of inflammation and physiological impairment (acidosis, hypothermia, coagulopathy) are important factors to consider when deeming patients eligible for DCS [173, 174].

## Conclusions

The acute abdomen after bariatric surgery is a common cause of admission in emergency departments. Knowledge of the most common late/long-term complications (> 4 weeks after surgical procedure) following LSG and LRYGB and their anatomy leads to a focused management in the emergency setting with good outcomes and decreased morbidity and mortality rates in a multidisciplinary approach.

**Table 1:** Summary of statements and recommendations of OBA guidelines

SUMMARY OF STATEMENTS AND RECOMMENDATIONS OF THE OPERATIVE MANAGEMENT OF ACUTE ABDOMEN AFTER BARIATRIC SURGERY (OBA) GUIDELINES
• ***PRIMARY ASSESSMENT***
**Q.1. WHICH ARE THE “ALARMING" CLINICAL SIGNS AND SYMPTOMS FOR ACUTE SURGICAL ABDOMEN IN PATIENTS WITH A PREVIOUS HISTORY OF BARIATRIC SURGERY?**
**Statement 1.1**
Tachycardia ≥ 110 beats per minute, fever ≥ 38 °C, hypotension, respiratory distress with tachypnea and hypoxia, and decreased urine output are alarming clinical signs in patients presenting with acute abdominal pain with a previous history of bariatric surgery **(QoE: low)**
**Statement 1.2**
In the presence of respiratory distress and hypoxia, a pulmonary embolism must be systematically excluded **(QoE: low)**
**Statement 1.3**
In the absence of fever and other signs of sepsis but in the presence of tachycardia (be aware of patients treated with beta blockers) and acute abdominal pain, patient requires immediate laboratory tests and imaging assessment for early and long-term complications following bariatric surgery **(QoE: low)**
**Statement 1.4**
In the emergency setting, the combination of fever, tachycardia, and tachypnea are significant predictors of an anastomotic leak or staple line leak after sleeve gastrectomy and Roux-en-Y gastric bypass **(QoE: low)**
**Statement 1.5**
Persisting vomiting and nausea are alarming clinical signs due to the high probability of complications such as internal hernia, volvulus, gastrointestinal stenosis, intestinal ischemia, or marginal ulcer after bariatric surgery **(QoE: low)**
**Statement 1.6**
The most common clinical presentation of internal hernia after laparoscopic Roux-en-Y gastric bypass is acute onset, persistent crampy/colicky abdominal pain, mostly located in the epigastrium **(QoE: low)**
**Statement 1.7**
The triad of persistent epigastric pain, pregnancy, and a history of laparoscopic Roux-en-Y gastric bypass should be warning signs for the prompt evaluation of the patient for the high suspicion of internal hernia **(QoE: low)**
**Statement 1.8**
Any clinical signs of intestinal bleeding such as hematemesis, melena, and hematochezia after bariatric surgery are predictors signs of intra-abdominal complications **(QoE: low)**
**Recommendation/1**
There are no absolute alarming clinical signs/symptoms for long-term complications after bariatric surgery. Clinical presentation can be non-specific. Any new onset abdominal symptoms should give rise to suspicion for long-term complications after bariatric surgery. We recommend against delaying prompt diagnostic work-up and laparoscopic surgical exploration in patients with a previous history of bariatric surgery, presenting with persistent abdominal pain and/or gastrointestinal symptoms, associated with fever, tachycardia, and tachypnea **(Strong recommendation based on low level of evidence 1C)**
**Q.2. WHICH ARE THE MOST SENSITIVE AND SPECIFIC LABORATORY TESTS FOR DIAGNOSIS IN PATIENTS WITH A PREVIOUS HISTORY OF BARIATRIC SURGERY PRESENTING WITH ACUTE ABDOMEN?**
**Statement 2.1** A detailed history, physical examination, laboratory tests, and imaging modalities are mandatory in decision-making algorithm for patients presenting with acute abdominal pain after a previous bariatric surgery, in the emergency setting **(QoE: low)**
**Statement 2.2**
Laboratory tests including complete blood count cells, serum electrolytes, C-reactive protein (CRP), procalcitonin, serum lactate levels, renal and liver function tests, serum albumin and blood gas analysis are helpful in the emergency department assessment of this group of patients presenting with acute abdominal pain **(QoE: low)**
**Statement 2.3**
High CRP level is predictive of both early and late postoperative complications after bariatric surgery **(QoE: low)**
**Statement 2.4**
CRP has a remarkably higher sensitivity and specificity than white blood count cells or neutrophil count to rule out an abdominal surgical disease. However a normal CRP level alone does not rule out the possibility of a postoperative complication following a bariatric surgical procedure **(QoE: low)**
**Statement 2.5**
Elevated serum lactates should not be used as a single marker to exclude internal herniation, because it can occur late in the presence of intestinal ischemia **(QoE: low)**
**Statement 2.6**
Nutritional deficiencies in vitamins, minerals, and trace elements may follow bariatric surgery and are associated with clinical manifestations and diseases, including anemia, ataxia, hair loss, and Wernicke encephalopathy **(QoE: low)**
**Recommendation/2**
There is not a biological marker for the diagnosis of long-term complications of bariatric surgery. We suggest performing a combination of complete blood count cells, serum electrolytes, serum albumin, liver and renal function tests, CRP, procalcitonin and serum lactate levels, blood gas analysis in assessing late complications following bariatric surgery in the emergency setting **(Weak recommendation based on low level of evidence 2C)** We suggest considering high CRP level and leukocytosis as predictors of abdominal emergencies following bariatric surgery **(Weak recommendation based on low level of evidence 2C)** We suggest assessing the nutritional status of patients undergoing bariatric procedures, including Vitamin D, folic acid, B12, B6, and B1 serum level, because of the high risk of vitamin B complex deficiency and malnutrition **(Weak recommendation based on low level of evidence 2C)**
**Q.3: WHICH IS THE MOST SPECIFIC AND SENSITIVE RADIOLOGICAL STUDY FOR DIAGNOSIS IN ASSESSING PATIENTS AFTER BARIATRIC SURGERY PRESENTING WITH ABDOMINAL PAIN?**
**Statement 3.1**
The diagnostic value of imaging after bariatric surgery depends mostly on the knowledge of the anatomic changes and of the potential complications following bariatric surgery **(QoE: low)**
**Statement 3.2**
Contrast-enhanced CT scan with oral contrast is the study of choice in patients with a previous history of bariatric surgery presenting with acute abdomen **(QoE: moderate)**
**Statement 3.3**
Plain abdominal X-ray has a limited role, when CT scan is not available, in detecting bowel distension or/and fluid levels **(QoE: low)**
**Statement 3.4**
Point-of-care ultrasound can be used by emergency physicians to rule out cholecystitis and biliary diseases, acute appendicitis, and the presence of free intraperitoneal fluid **(QoE: low)**
**Statement 3.5**
The administration of oral and intravenous contrast is fundamental to find landmarks for the interpretation of images **(QoE: low)**
**Statement 3.6**
In a pregnant woman with a history of bariatric surgery, US and magnetic resonance imaging (MRI) are preferred to assess acute abdominal pain with the aim of limiting ionizing radiation exposure. Low-dose CT could be performed in very selected cases **(QoE: low)**
**Statement 3.7**
Diagnostic laparoscopy has higher sensitivity and specificity than any radiological assessment **(QoE: moderate)**
**Statement 3.8**
The role of angiography and angioembolization in patients presenting with a gastrointestinal bleeding after bariatric surgery is marginal. They could be a valid tool to achieve bleeding control, in selected cases **(QoE: very low)**
**Recommendations/3**
We recommend the use of contrast-enhanced computed tomography with oral contrast in the assessment of acute abdomen after bariatric surgery, whenever possible. The absence of oral and intravenous contrast can significantly decrease sensitivity and specificity of radiological assessment **(Strong recommendation based on low level evidence 1C)** We recommend assessing the acute abdomen in a pregnant woman by US and MRI to limit radiation exposure, though low-dose CT can be useful in selected cases **(Strong recommendation based on low level evidence 1C)** We recommend against delaying laparoscopic exploration if there is a high index of clinical suspicion and in the presence of alarming clinical signs/symptoms, even in situations of negative radiological assessment **(Strong recommendation based on low level evidence 1C)**

• ***PREOPERATIVE AND NON-OPERATIVE MANAGEMENT***
**Q.4: WHAT IS THE ROLE OF ENDOSCOPY IN THE DIAGNOSIS AND TREATMENT OF LONG-TERM COMPLICATIONS FOLLOWING BARIATRIC SURGERY IN THE EMERGENCY SETTING?**
**Statement 4.1**
After contrast-enhanced computed tomography with oral contrast administration, the endoscopic evaluation is the first tool to be considered for the diagnosis and management of leaks and fistulae related to bariatric surgery in stable patients **(QoE: moderate)**
**Statement 4.2**
The endoscopic evaluation should be performed by an expert endoscopist aware of the new anatomy resulting from different surgical bariatric procedures **(QoE: moderate)**
**Statement 4.3**
The endoscopic management of leaks and fistulae related to bariatric surgery in stable patients is effective and safe when performed in expert centers **(QoE: moderate**)
**Statement 4.4**
Several endoscopic devices and techniques are available to manage bariatric surgery complications and they include internal drainage techniques and vacuum therapy, self-expanding metal (SEMS) and plastic stents, clipping techniques, including the use of through-the-scope clips (TTSC) and over-the-scope clips (OTSC), tissue sealants, suturing systems (OverStitch System®) **(QoE: moderate)**
**Statement 4.5**
The endoscopic internal drainage by pigtail plastic stents is considered an effective alternative to fully covered SEMS in the treatment of late leaks and fistulae after LSG or RYGB providing higher success rates, shorter treatment duration, and lower adverse events rates compared to stenting **(QoE: moderate)**
**Statement 4.6**
In the presence of gastric stricture after LSG or of anastomotic stricture after LRYGB, the endoscopic dilation performed by achalasia balloon in LSG or through-the-scope dilation hydrostatic balloon for LRYGB should be considered as the first-line treatment **(QoE: low)**
**Statement 4.7**
Early endoscopic assessment should be performed in case of suspected active intra-gastric bleeding related to a marginal or stomal ulcer in stable patients. **(QoE: low)**
**Statement 4.8**
Hemostasis of a bleeding ulcer, performed with injection of epinephrine or mechanical hemostasis with endoscopic clips and rubber band ligation are preferred over thermal hemostasis **(QoE: moderate)**
**Statement 4.9**
Novel hemostatic powders may be considered as a valid therapeutic option in selected patients presenting with a bleeding ulcer **(QoE: moderate)**
**Recommendation/4**
In the high suspicion and diagnosis of staple line leak, gastric fistula, delayed gastro-jejunal anastomotic leakage, marginal or stomal ulceration, gastro-gastric fistula, gastro-jejunal anastomotic stricture and intra-luminal bleeding after LSG and LRYGB, we recommend an urgent endoscopic assessment and management in hemodynamically stable patients, according to the availability of an endoscopist with expertise in bariatric surgery. We recommend against delaying surgical exploration in hemodynamically unstable patients **(Strong recommendation based on low level of evidence 1C)**
**Q.5: HOW SHOULD ANTIBIOTICS BE USED IN THE MANAGEMENT OF THE ACUTE ABDOMEN IN PATIENTS WITH PREVIOUS HISTORY OF BARIATRIC SURGERY?**
**Statement 5.1**
The optimal management of patients presenting with sepsis requires early source control, adequate empiric antimicrobial therapy, and targeted fluid resuscitation **(QoE: moderate)**
**Statement 5.2**
Complicated IAIs are multi bacterial mainly caused by Gram-negative bacilli and anaerobes. Broad-spectrum single-agent or combination drug regimens targeting these microorganisms are the mainstay of early empiric antimicrobial therapy **(QoE: moderate)**
**Statement 5.3**
Obesity alone is not associated with antimicrobial treatment failure among patients with IAI. Obesity may not be an absolute indication for longer duration of antimicrobial therapy in treatment of IAI **(QoE: moderate)**
**Statement 5.4**
A short (3–4 days of IV antibiotics) course of therapy may be effective even in critically ill surgical patients with complicated IAI, including fungal infection, after adequate source control **(QoE: moderate)**
**Statement 5.5**
The empiric implementation of antifungal therapy due to perforated marginal ulcer (ulceration located on the jejunal side of the anastomosis) or stomal ulcer (ulceration on the gastric side) is not supported by the literature in community-acquired IAI **(QoE: moderate)**
**Statement 5.6**
The dose of antibiotics must be adjusted to the weight and renal function of the patient **(QoE: high)**
**Recommendations/5**
We recommend administering early empiric broad-spectrum antimicrobial therapy in patients presenting with intra-abdominal infections, in addiction to adequate source control. After surgical management, short-term antimicrobial therapy is preferred even in critically ill patients **(Strong recommendation based on moderate quality of evidence 1B)** We suggest administering antifungal therapy in frail, immunocompromised patients, presenting with biological and clinical signs of sepsis, and if fungal organisms are isolated in the intraperitoneal fluid **(Weak recommendation based on low quality of evidence 2C)**
**Q.6: HOW CAN WE DECREASE THE RISK OF THROMBOEMBOLIC EVENTS IN SURGICAL PATIENTS WITH A PREVIOUS HISTORY OF BARIATRIC PROCEDURES AFTER EMERGENCY SURGERY?**
**Statement 6.1**
All patients needing an urgent surgical procedure should be risk stratified for venous thromboembolism **(QoE: moderate)**
**Statement 6.2**
In the emergency setting, venous thromboembolism pharmacologic prophylaxis should be started as soon as possible, if there are no signs of active bleeding, to decrease the risk of venous thromboembolism **(QoE: moderate)**
**Statement 6.3**
If contraindications to pharmacologic venous thromboembolism prophylaxis exist, mechanical venous thromboembolism prevention strategies such as compression stockings and foot pump should be considered even if the patient is ambulating, because of the high venous thromboembolism risk following emergency surgery **(QoE: low)**
**Statement 6.4**
Pharmacologic venous thromboembolism prophylaxis with unfractionated heparin or low-molecular-weight heparin is highly effective for the prevention of venous thromboembolism in hospitalized patients **(QoE: moderate)**
**Statement 6.5**
The venous thromboembolism prophylaxis should continue after discharge according to the thrombotic risk. This can be estimated based on patient- and procedure-specific factors, applying validated risk assessment models such as the Caprini score. Patients with a prolonged hospital stay, the presence of cancer, urinary tract infection, and postoperative sepsis are at high risk to present with venous thromboembolism **(QoE: moderate)**
**Recommendation/6**
We recommend the administration of low-molecular-weight heparin for venous thromboembolism prophylaxis as soon as possible in patients presenting with an acute surgical abdomen after bariatric surgery**.** The LMWH dose should be adjusted to the patient’s weight, the thrombotic risk and creatinine clearance **(Strong recommendation based on moderate level of evidence 1B)** We suggest to continue venous thromboembolism prophylaxis at least 4 weeks after discharge **(Weak recommendation based on moderate level evidence 2B)** We suggest to use the monitoring of anti-Xa levels to adjust LMWH dose, particularly in elderly, pregnant, renally impaired, with BMI = > 35 kg/m2 patients, at risk of suboptimal dosing **(Weak recommendation based on moderate level of evidence 2B)** In patients where pharmacologic venous thromboembolism prophylaxis is contraindicated, we recommend the use of mechanical prophylaxis, especially in high-risk patients **(Strong recommendation based on moderate level evidence 1B)**

• ***OPERATIVE MANAGEMENT***
**Q.7: WHICH SURGICAL PROCEDURES ARE EFFECTIVE IN THE MANAGEMENT OF ACUTE ABDOMEN FOLLOWING BARIATRIC SURGERY?**
**Scenario 1: BLEEDING**
**Statement 7.1**
Intra-abdominal bleeding occurs very rarely as a late complication after bariatric surgery **(QoE: low)**
**Statement 7.2**
The most common reason for late gastrointestinal bleeding is ulcer from gastric sleeve and gastro-jejunostomy, gastric pouch, bypassed stomach, or duodenum after LRYGB **(QoE: moderate)**
**Statement 7.3**
Bleeding after LRYGB and LSG is self-limiting in many patients and can be managed conservatively, with medical treatment and close monitoring and observation **(QoE: low)**
**Statement 7.4**
In hemodynamic stable patients presenting with intra-luminal intestinal bleeding after bariatric surgery, the endoscopic assessment (that must be performed after endotracheal intubation to protect airways) is a valid and safe tool after bariatric surgery **(QoE: moderate)**
**Statement 7.5**
Angiography and angioembolization may be valid strategies to bleeding control **(QoE: very low)**
**Statement 7.6**
If endoscopic and angiographic management fail, surgical exploration is indicated, if there are signs of persisting bleeding **(QoE: low)**
**Statement 7.7**
In unstable patients not responding to aggressive resuscitation, diagnostic laparotomy and surgical hemostasis are mandatory **(QoE: high)**
**Recommendations/7-BLEEDING**
Endoscopy is the first recommended diagnostic tool in stable patients presenting with gastrointestinal bleeding after SG and RYGB **(Strong recommendation based on moderate quality of evidence 1B)** We suggest performing an angio-CT and embolization in stable patients presenting with gastrointestinal and intraperitoneal extra-luminal bleeding, when skills are available **(Weak recommendation based on low level of evidence 2C)** In selected cases of hemodynamically stable bleeding peptic ulcer patients, after failure to attempt endoscopic hemostasis, we suggest the use of angiography with angioembolization if technical skills and equipment are available **(Weak recommendation based on very low level of evidence 2D)** We recommend against delaying surgical exploration in unstable patients presenting with ongoing gastrointestinal bleeding after endoscopic assessment and negative angio-CT scan for bleeding source localization **(Strong recommendation based on low level evidence 1C)** In patients selected for surgical exploration for a bleeding ulcer, we suggest planning intra-operative endoscopy to facilitate the localization of the bleeding site, using a surgical gastrostomy in case of patient with LRYGB to assess the gastric remnant and duodenum **(Weak recommendation based on very low quality of evidence, 2D)** We recommend against delaying diagnostic laparoscopy/laparotomy in patients presenting with ongoing intraperitoneal, extra-luminal bleeding, after angioembolization **(Strong recommendation based on low level of evidence 1C)** A biopsy of the bleeding ulcer is recommended to exclude malignancy **(Strong recommendation based on low level of evidence, 1C)**
**Scenario 2: INTESTINAL OBSTRUCTION**
**Statement 7.8**
Stenosis after LSG in stable patients presenting with gastrointestinal symptoms should be assessed by endoscopy and the treatment must be tailored according to the clinical status of the patient and endoscopic findings **(QoE: low)**
**Statement 7.9**
Endoscopic pneumatic dilation is a safe and effective first-line treatment of gastric stenosis after SG and gastro-jejunostomy strictures after RYGB. Perforation is a potential complication of this treatment and may necessitate surgical intervention **(QoE: moderate)**
**Statement 7.10**
After RYGB, the most common causes of small-bowel obstruction (SBO) are internal hernia, adhesions, incisional hernia/trocar site hernia, intussusception, volvulus, obstruction localized at the jejuno-jejunostomy (JJ), twisted alimentary limb, alimentary limb mesenteric ischemia, and adhesions proximal to the JJ **(QoE: low)**
**Statement 7.11**
In case of SBO after RYGB, an exploratory laparoscopy is mandatory in the first 12–24 h in stable patients presenting with persistent abdominal pain and inconclusive clinical and radiological findings **(QoE: low)**
**Statement 7.12**
Diagnostic laparoscopy in pregnant women with SBO after bariatric surgery is effective and is associated with a good maternal and fetal outcome **(QoE: very low)**
**Statement 7.13**
Surgical exploration in patients after LRYGB should start from the ileocecal junction (distally to the obstruction) toward the inspection of the jejuno-jejunostomy and of the 3 potential site locations of internal hernia: the traverse mesocolon (in retrocolic bypasses), Petersen’s space (Petersen’s hernia) and the jejuno-jejunostomy mesenteric defect (mesojejunal hernia), and then the remnant stomach. **(QoE: very low)**
**Statement 7.14**
If an internal hernia is found, an assessment of intestinal viability should be undertaken; if intestinal ischemia is present, surgical resection is performed. The closure of the mesenteric defect should be performed with non-absorbable material in running or interrupted suture **(QoE: very low)**
**Statement 7.15**
Indocyanine green (ICG) fluorescence angiography may be a valid tool in the evaluation of the extent of bowel resection and anastomosis perfusion, when it is available **(QoE: very low)**
**Statement 7.16**
If no internal hernia or other evident causes of SBO are found, the entire small intestine should be assessed given that there are other causes of intestinal obstruction (adhesions, intussusception, volvulus) **(QoE: very low)**
**Statement 7.17**
In case of intra-operative findings of intussusception, the surgical treatment may be limited to reduction if the small bowel is viable, but resection of the affected segment is recommended since it seems to result in fewer recurrences **(QoE: very low)**
**Statement 7.18**
In the emergency setting, when SBO is due to a bezoar located in the stomach, the first approach could be endoscopy. If the bezoar is located distally in the small bowel, surgical intervention is required to milk the bezoar into the cecum or remove it by creating an enterotomy **(QoE: very low)**
**Recommendations/7-INTESTINAL OBSTRUCTION**
In the presence of symptoms of proximal SBO after LSG and LRYGB, endoscopic assessment is recommended in stable patients **(Strong recommendation based on low level of evidence 1C)** In the presence of SBO after RYGB, we recommend performing an exploratory laparoscopy in the first 12–24 h in stable patients with a history of bariatric surgery presenting with persisting acute abdominal pain after inconclusive laboratory, radiological and endoscopic results in the emergency setting (**Strong recommendation based on low level of evidence 1C)** We suggest considering limited intestinal resection and anastomosis in case of clear intestinal segmental ischemia in hemodynamically stable patients, or damage control and open abdomen approach in cases of extended intestinal ischemia/peritonitis in hemodynamic unstable patients **(Weak recommendation based on low level evidence 2C)**
**Scenario 3: Localized or Generalized PERITONITIS**
**Statement 7.19**
Stable patients with perforated gastro-jejunal ulcer after LRYGB should be managed with laparoscopic primary repair by suturing and omental patch which are safe and feasible and associated with decreased operative time, blood loss, and length of stay **(QoE: low)**
**Statement 7.20**
In patients presenting with marginal ulceration and perforation at the jejuno-jejunal anastomosis, laparoscopic primary suturing is a valid option in selected patients (young patients, early presentation, no other serious comorbidities, hemodynamic stability) **(QoE: low)**
**Statement 7.21**
If a gastro-gastric fistula is found at surgical exploration for a perforated ulcer, surgical options include simple resection of the fistula, resection of the fistula with revision of the gastro-jejunal anastomosis (GJA), resection of the fistula with remnant gastrectomy ± revision of GJA, or gastrectomy of the remnant stomach (**QoE: very low)**
**Statement 7.22**
After LRYGB, a perforated remnant stomach may be managed by primary suture and omental patch or stapled resection, If there is concern for significant postoperative ileus due to peritonitis, a gastrostomy tube could be placed in the gastric remnant proximal to the site of the perforation to decompress the stomach and allow a postoperative endoscopic access **(QoE: low)**
**Statement 7.23**
If diffuse peritonitis is due to a perforated excluded gastrointestinal segment (stomach or duodenum), it is recommended to explore the jejuno-jejunostomy (stenosis) or the gastric remnant (gastro-gastric fistula) **(QoE: low)**
**Statement 7.24**
Surgical treatment of a duodenal perforation depends on the hemodynamic stability of the patient, size of the perforation and extent of native tissue loss **(QoE: very low)**
**Recommendations/7-PERITONITIS**
We recommend performing immediate surgical exploration in unstable patients presenting with peritonitis without delay (**Strong recommendation based on low level evidence 1C)** We recommend assessing all anastomoses after LRYGB, the remnant stomach and the excluded duodenum (**Strong recommendation based on low level evidence 1C)** We recommend performing biopsies of the perforated ulceration to exclude malignancy (**Strong recommendation based on low level evidence 1C)** We recommend performing primary suture with omental patch in laparoscopic approach in stable patients presenting with a perforated marginal ulcer or gastric remnant or duodenal perforation of less than1cm, whenever technically possible **(Strong recommendation based on low level evidence 1C)** We suggest considering damage control surgery and open abdomen in hemodynamically unstable patients **(Weak recommendation based on low level evidence 2C)**
**Q.8: WHAT IS THE ROLE OF DAMAGE CONTROL SURGERY IN THE MANAGEMENT OF PATIENTS WITH ACUTE ABDOMEN DUE TO LATE COMPLICATIONS OF BARIATRIC SURGERY?**
**Statement 8.1**
Damage control surgery may be a tool to consider in the management of the acute abdomen in patients presenting with persistent hemodynamic instability because of severe peritonitis and septic shock **(QoE: low)**
**Statement 8.2**
The open abdomen is an option for emergency surgery patients with severe peritonitis and severe sepsis/septic shock in the context of an abbreviated laparotomy due to severe physiological derangement, the need for a deferred intestinal anastomosis, a planned second look for intestinal ischemia, persistent source of peritonitis (failure of source control), or extensive visceral edema with concerns for the development of abdominal compartment syndrome **(QoE: low)**
**Recommendation/8**
We suggest the use of damage control surgery with open abdomen in hemodynamic unstable patients secondary to an intra-abdominal source of infection, to extensive intestinal ischemia and massive hemoperitoneum **(Weak recommendation based on low level evidence 2C)**

## Data Availability

Not applicable.

## Abbreviations

US: Ultrasonography
CT: Computed tomography
MRI: Magnetic resonance imaging
GI: Gastrointestinal
LMWH: Low-molecular-weight heparin
WSES: World Society of emergency surgery
IH: Internal hernia
AAP: Acute abdominal pain
LSG: Laparoscopic sleeve gastrectomy
LRYGB: Laparoscopic Roux-en-Y gastric bypass
CRP: C-reactive protein
WBC: White blood count cells
IV: Intravenous
GJ: Gastro-jejunostomy
SBO: Small-bowel obstruction
PPU: Perforated peptic ulcer
IAI: Intra-abdominal infection
EID: Endoscopic internal drainage
FC-SEMS: Full-covered self-expandable metallic stent
EVT: Endoscopic vacuum therapy
