# Correction: Operative management of acute abdomen after bariatric surgery in the emergency setting: the OBA guidelines

**DOI:** 10.1186/s13017-022-00460-w

**Published:** 2022-11-07

**Authors:** Belinda De Simone, Elie Chouillard, Almino C. Ramos, Gianfranco Donatelli, Tadeja Pintar, Rahul Gupta, Federica Renzi, Kamal Mahawar, Brijesh Madhok, Stefano Maccatrozzo, Fikri M. Abu-Zidan, Ernest E. Moore, Dieter G. Weber, Federico Coccolini, Salomone Di Saverio, Andrew Kirkpatrick, Vishal G. Shelat, Francesco Amico, Emmanouil Pikoulis, Marco Ceresoli, Joseph M. Galante, Imtiaz Wani, Nicola De’Angelis, Andreas Hecker, Gabriele Sganga, Edward Tan, Zsolt J. Balogh, Miklosh Bala, Raul Coimbra, Dimitrios Damaskos, Luca Ansaloni, Massimo Sartelli, Nikolaos Pararas, Yoram Kluger, Elias Chahine, Vanni Agnoletti, Gustavo Fraga, Walter L. Biffl, Fausto Catena

**Affiliations:** 1 Department of Emergency, Digestive and Metabolic Minimally Invasive Surgery, Poissy and Saint Germain en Laye Hospitals, Poissy-Ile de France, France; 2 GastroObesoCenter Institute for Metabolic Optimization, Sao Paulo, Brazil; 3 Interventional Endoscopy and Endoscopic Surgery, Hôpital Privé Des Peupliers, Paris, France; 4 Department of Abdominal Surgery, Ljubljana University Medical Centre, Ljubljana, Slovenia; 5 Division of Minimally Invasive Surgery and Bariatrics, Beth Israel Deaconess Medical Center, Boston, MA USA; 6 General Surgery and Trauma Team, ASST Niguarda, Piazza Ospedale Maggiore 3, 20162 Milano, Milan Italy; 7 South Tyneside and Sunderland NHS Foundation Trust, Sunderland, UK; 8 East Midlands Bariatric and Metabolic Institute, University Hospitals of Derby and Burton NHS Trust, Derby, UK; 9 Department of Bariatric Surgery, Istituto Di Cura Beato Matteo, Vigevano, Italy; 10 Department of Surgery, College of Medicine and Health Sciences, UAE University, Al-Ain, United Arab Emirates; 11 Denver Health System - Denver Health Medical Center, Denver, USA; 12 Department of General Surgery, Royal Perth Hospital, University of Western Australia, Perth, Australia; 13 Department of Emergency and Trauma Surgery, Pisa University Hospital, Pisa, Italy; 14 Department of Surgery, Madonna Del Soccorso Hospital, San Benedetto del Tronto, Italy; 15 Department of General, Acute Care, Abdominal Wall Reconstruction, and Trauma Surgery, Foothills Medical Centre, Calgary, AB Canada; 16 Department of General Surgery, Tan Tock Seng Hospital, Singapore, Singapore; 17 Department of Surgery, John Hunter Hospital and The University of Newcastle, Newcastle, MSW Australia; 18 3Rd Department of Surgery, Attikon General Hospital, National and Kapodistrian University of Athens (NKUA), Athens, Greece; 19 General Surgery, Monza University Hospital, Monza, Italy; 20 University of California, Davis 2315 Stockton Blvd., Sacramento, CA 95817 USA; 21 Government Gousia Hospital, Srinagar, Kashmir India; 22 Service de Chirurgie Digestive Et Hépato-Bilio-Pancréatique - DMU CARE, Hôpital Henri Mondor, Paris, France; 23 Department of General and Thoracic Surgery, University Hospital of Giessen, Giessen, Germany; 24 Emergency Surgery and Trauma, Fondazione Policlinico Universitario A. Gemelli IRCCS, Università Cattolica del Sacro Cuore, Rome, Italy; 25 Department of Emergency Medicine, Radboud University Medical Center, Nijmegen, The Netherlands; 26 Department of Traumatology, John Hunter Hospital and University of Newcastle, Newcastle, NSW Australia; 27 Trauma and Acute Care Surgery Unit, Hadassah - Hebrew University Medical Center, Jerusalem, Israel; 28 Riverside University Health System Medical Center, Loma Linda University School of Medicine, Riverside, CA USA; 29 General and Emergency Surgery, Royal Infirmary of Edinburgh, University of Edinburgh, Edinburgh, UK; 30 Department of Surgery, Pavia University Hospital, Pavia, Italy; 31 Department of General Surgery, Macerata Hospital, Macerata, Italy; 32 Division of General Surgery, Rambam Health Care Campus, Haifa, Israel; 33 Department of Emergency and Trauma Surgery, Bufalini Hospital, Cesena, Italy; 34 School of Medical Sciences, University of Campinas (Unicamp), Campinas, SP Brazil; 35 Department of Trauma and Acute Care Surgery, Scripps Memorial Hospital La Jolla, La Jolla, San Diego, CA USA

**Correction: World Journal of Emergency Surgery (2022) 17:51** https://doi.org/10.1186/s13017-022-00452-w

Following publication of the original article [1], the co-author “Nikolaos Pararas” has misspelled wrongly as “Nikolaos Parasas”.

The original article has been corrected.
